# Developmental Hypoxia Enhances Kidney Organoid Complexity and Maturity

**DOI:** 10.1002/advs.202501661

**Published:** 2025-08-21

**Authors:** Hyeonji Lim, Dohui Kim, Haejin Yoon, Joo H. Kang, Yong Jun Kim, Dong Sung Kim, Tae‐Eun Park

**Affiliations:** ^1^ Department of Biomedical Engineering College of Information‐Bio Convergence Engineering Ulsan National Institute of Science and Technology (UNIST) Ulsan 44919 Republic of Korea; ^2^ Department of Mechanical Engineering Pohang University of Science and Technology (POSTECH) Pohang 37673 Republic of Korea; ^3^ Department of Biological Sciences College of Information‐Bio Convergence Engineering Ulsan National Institute of Science and Technology (UNIST) Ulsan 44919 Republic of Korea; ^4^ Department of Pathology College of Medicine Kyung Hee University Seoul 02447 Republic of Korea; ^5^ Department of Chemical Engineering Pohang University of Science and Technology (POSTECH) Pohang 37673 Republic of Korea; ^6^ School of Interdisciplinary Bioscience and Bioengineering Pohang University of Science and Technology (POSTECH) Pohang 37673 Republic of Korea; ^7^ Institute for Convergence Research and Education in Advanced Technology Yonsei University Seoul 03722 Republic of Korea

**Keywords:** human induced pluripotent stem cell, hypoxia, kidney organoid, nephrogenesis, polycystic kidney disease

## Abstract

Reciprocal signaling between metanephric mesenchyme (MM) and ureteric bud (UB) is essential for human kidney development. However, human pluripotent stem cell‐derived kidney organoids do not incorporate UB differentiation, limiting organoid maturation and disease modeling. Here, a hypoxia‐based differentiation method inspired by developmental cues is reported that produces mature kidney organoids with collecting duct‐like tubules connected to multiple nephrons. Hypoxia promotes the co‐induction of MM and UB‐like progenitors within the same culture dish. The augmented expression of reciprocal signaling genes guides the differentiation of the kidney organoids into highly structured tubular networks, mature RNA profiles, and a more realistic micro‐anatomy, leading to higher‐order kidney organogenesis in vitro. These hypoxia‐enhanced kidney organoids recapitulate the cystic phenotype in polycystic kidney disease, displaying efficient cyst formation across the entire tubular region and increased sensitivity to drugs. The findings provide an improved in vitro model for studying kidney development and disease mechanisms.

## Graphical abstract

The current bottleneck in metanephric mesenchyme (MM)‐derived nephron organoid development is the absence of a connected network of collecting ducts (CD) derived from the ureteric bud (UB). To address this limitation, this study proposes a hypoxia‐based differentiation method that mimics the natural renal developmental environment, enabling the generation of mature kidney organoids that replicate the in vivo renal structure.

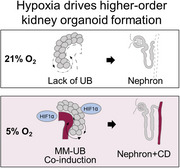

## 1 Introduction

Kidney development involves intricate interactions between the ureteric bud (UB), derived from the anterior intermediate mesoderm (aIM), and the metanephric mesenchyme (MM), from the posterior intermediate mesoderm (pIM).^[^ ^1^ ^]^ Signals from the MM induce UB branching, forming the collecting ducts, while UB signals drive nephron formation in the MM.^[^ ^2^ ^]^ This interplay is essential for creating functional kidneys in vitro, where nephrons are connected to collecting ducts (CD).

Current kidney organoid protocols focus mainly on generating MM‐derived nephron progenitor cells (NPCs), including podocytes, Bowman's capsule parietal epithelial cells, and renal tubular epithelial cells, but often neglect UB‐derived cells.^[^ ^3^ ^]^ For example, the Morizane protocol uses prolonged WNT signaling and Activin A to induce NPCs, based on the knowledge that pIM migrates out of the primitive streak (PS) later than does aIM,^[^ ^4^ ^]^ thereby being exposed to long WNT signaling in the PS.^[^ ^5^ ^]^ Although this selective induction of NPCs enables efficient generation of the complex structure of human nephrons in vitro, the formed nephrons are separately positioned within the organoids, and their organization and function do not mimic those of a mammalian kidney due to the lack of UB‐derived cells and signals. Similarly, the Takasato protocol produces both MM‐ and UB‐derived cells by modulating WNT, FGF9, and retinoic acid exposure, but the UB‐like cells resemble distal renal tubules, not collecting ducts.^[^ ^3a^ ^]^ As a result, these organoids are limited in replicating the pathogenesis of UB‐derived structures, such as polycystic kidney disease (PKD), which involves the presence of renal cysts throughout all segments of the renal tubules.^[^ ^3^, ^6^ ^]^

To address these limitations, researchers have explored strategies for generating UB cells from mouse/rat tissue or human pluripotent stem cells (hPSCs) and assembling them with NPCs, replicating reciprocal interactions between UB and MM during kidney organogenesis, resulting in higher‐order kidney structures. However, this approach requires animal‐derived stromal cells for organoid maturation^[^ ^7^ ^]^ and lacks vascular structures,^[^ ^7^, ^8^ ^]^ making it labor‐intensive.

In this study, we demonstrate that replicating low oxygen tension during the early stages of kidney differentiation enables the differentiation of human induced pluripotent stem cells (hiPSCs) into MM and UB‐like cells within the same culture dish. This simultaneous induction of these progenitor cells enhances the expression of mutual signaling genes, such as WNTs, promoting the development of well‐structured tubules that closely resemble the architecture of an in vivo kidney, including UB‐derived collecting duct‐like tubules connected to multiple MM‐derived nephrons. Our unique approach not only improves the kidney organoid maturation without the need for complex experimental procedures but also enables more accurate disease modeling and drug testing.

## 2 Results

### 2.1 The Impact of Hypoxic Stimulation on the Commitment of Renal Progenitors

**Figure 1:**
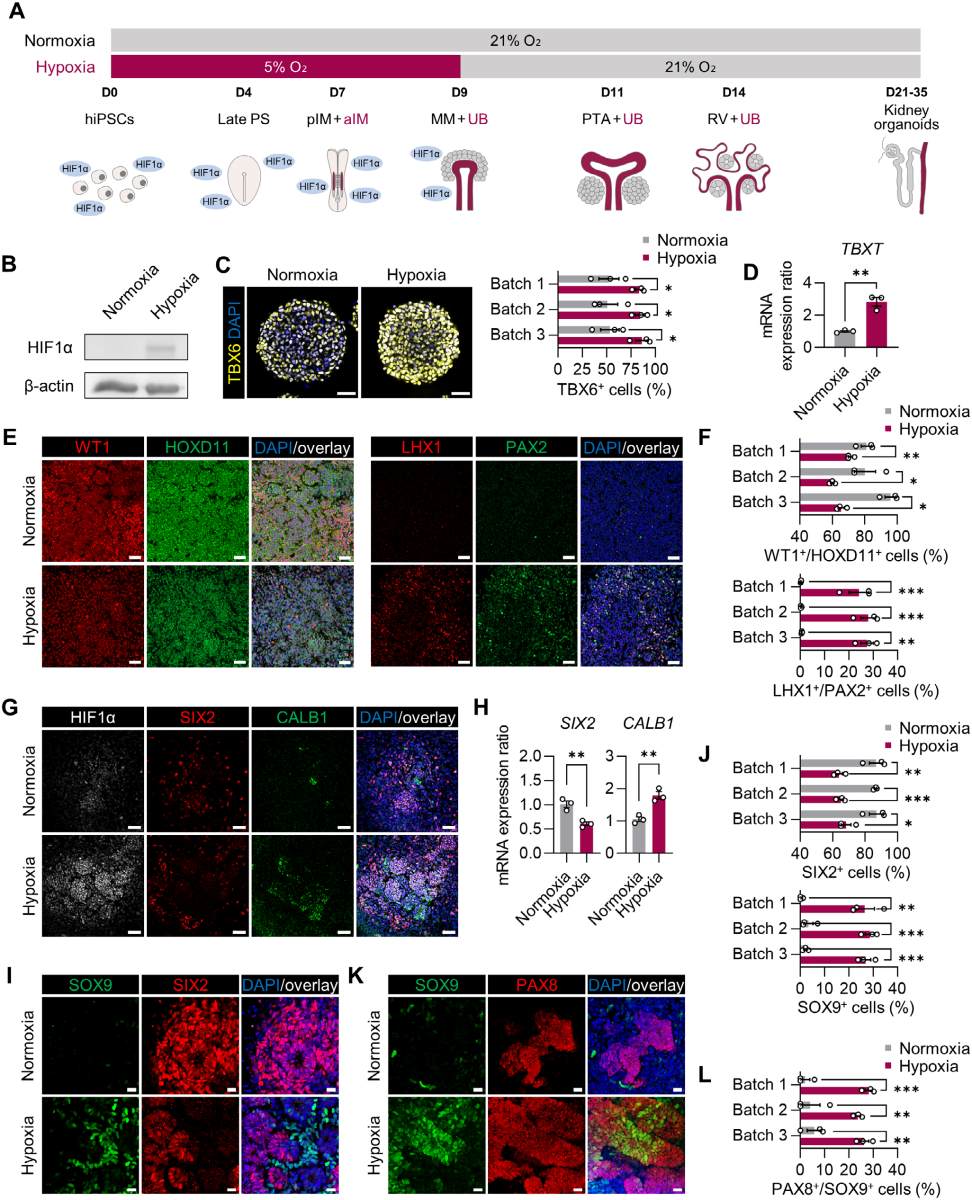
Dual induction of MM and UB‐like cells from hiPSCs under hypoxic stimulation. A) Timeline for differentiation of the kidney organoids from hiPSCs under normoxic and hypoxic conditions. hiPSCs, human induced pluripotent stem cells; PS, primitive streak; pIM, posterior intermediate mesoderm; aIM, anterior intermediate mesoderm; MM, metanephric mesenchyme; UB, ureteric bud; PTA, pre‐tubular aggregate; RV, renal vesicle. B) Western blot showing HIF1α expression on day 1 under normoxic and hypoxic conditions. C) Left: Immunofluorescent micrographs at day 4 labeled with PS marker (TBX6). Scale bars, 50 µm. Right: The percentage of TBX6^+^ cells on day 4 in hypoxia and normoxia. D) qRT‐PCR on day 4 showing the expression of a PS marker (*TBXT*) in cells cultured under hypoxic versus normoxic conditions. E) Left: Immunofluorescence micrographs of the cells on day 7 of differentiation labeled with pIM markers (WT1 and HOXD11) and aIM markers (LHX1 and PAX2). Scale bars, 50 µm. F) The percentage of co‐expressed cells positive for WT1^+^/HOXD11^+^ and LHX1^+^/PAX2^+^ analyzed by Image J. G,I) Fluorescence microscopy images of the cells on day 9 of differentiation under normoxic and hypoxic conditions, stained for HIF1α, MM marker (SIX2), and UB marker (CALB1 and SOX9). Scale bars, 50 µm (G) and 20 µm (I). H) qRT‐PCR on day 9 showing relative expression of *SIX2* and *CALB1* under the hypoxic and normoxic conditions. J) The percentage of SIX2^+^ and SOX9^+^ cells on day 9 under normoxia and hypoxia. K) Immunofluorescent micrographs of the cells on day 14 of differentiation in the normoxic and hypoxic conditions, stained for RV marker (PAX8), and UB marker (PAX8 and SOX9). Scale bars, 20 µm. L) The percentage of PAX8^+^/SOX9^+^ co‐expressing cells differentiated under normoxia and hypoxia on day 14. All data are plotted as mean ± S.E. and *N* = 3 for the independent experiments. (C,D,F,H,J,L) *P* values were determined by two‐tailed unpaired *t*‐test (^*^ *p* < 0.05; ^**^ *p* < 0.01; ^***^ *p* < 0.001).

Hypoxic stimulation can promote the commitment of progenitors to various cell types, and microenvironmental oxygen concentration is an important signal for regulating embryo development.^[^ ^9^ ^]^ Previous in vivo and in vitro research has underscored the role of HIF1α in the development of kidneys, suggesting its regulatory function in early nephrogenesis.^[^ ^10^ ^]^ To explore the possibility that oxygen regulation could improve the formation of organized kidney structures, we modified the Morizane protocol^[^ ^3c^ ^]^ by shifting the culture to hypoxic conditions (5% O_2_) for 9, 14, and 21 days, corresponding to MM, renal vesicle (RV), and nephron stages, respectively (Figure S1A, Supporting Information). Kidney organoid differentiation of hiPSC line (IMR90‐4) under 5% O_2_ for 9 days and 21% O_2_ for the next 12 days resulted in the highest induction of tubular structures, which showed distinct morphological differences from organoids cultured according to the original Morizane protocol (“Normoxia”) (**Figure** **1**A; Figure S1A, Supporting Information). Cells exposed to 1% instead of 5% O_2_ for 9 days did not produce self‐organized renal structures (Figure S1A, Supporting Information), suggesting that oxygen can stimulate kidney differentiation under precise levels and temporal dynamics of oxygen tension.^[^ ^10a^ ^]^ We further investigated whether the optimized hypoxic conditions (“Hypoxia”; 5% O_2_ for 9 days) stimulated the differentiation of hiPSCs into UB and MM.

Following the Morizane protocol, we treated cells with CHIR and Noggin as a WNT activator and a BMP inhibitor for four days to induce TBX6^+^/TBXT^+^ PS formation under normoxic or hypoxic conditions.^[^ ^3c^ ^]^ Western blot analysis revealed that hypoxia‐inducible transcription factor 1α (HIF1α) protein level was upregulated when exposed to hypoxia during differentiation (Figure 1B). The hypoxic conditions (84.60 ± 0.85%) robustly stimulated PS induction, consistently demonstrating greater efficiency than the normal conditions (52.12 ± 0.76%) across the various batches (Figure 1C). A statistically significant increase in mRNA expression of *TBXT* was observed in cells on day 4 under hypoxic versus normoxic conditions (Figure 1D). This is consistent with recent findings that early mesoderm and endoderm‐instructive genes, including *TBXT*, are selectively upregulated in PSCs under hypoxia, and that this regulation is mediated by HIF1α.^[^ ^11^ ^]^

Subsequent treatment with Activin A between days 4 and 7, as per a previous study,^[^ ^3c^ ^]^ induced formation of pIM cells as indicated by WT1 and HOXD11 markers. We observed substantial populations of WT1^+^/HOXD11^+^ co‐expressing cells in both normoxia and hypoxia (Figure 1E; Figure S1B,C, Supporting Information); however, the proportion of WT1^+^/HOXD11^+^ cells in hypoxia (65.56 ± 3.21%) was consistently found to be lower than under normoxia (85.71 ± 5.11%) across the different batches (Figure 1F; Figure S1C, Supporting Information). On the other hand, we observed a remarkable increase in cells co‐expressing LHX1^+^/PAX2^+^ (Figure 1E), which are markers of aIM.^[^ ^3c^ ^]^ The percentage of the LHX1^+^/PAX2^+^ cells was significantly increased under hypoxia (26.53 ± 1.18%) compared to normoxia (0.41 ± 0.10%) (Figure 1E,F), indicating that hypoxia facilitated the differentiation of hiPSCs into LHX1^+^/PAX2^+^ aIM‐like cells, the precursors to the UB.

By applying FGF9 from days 7 to 9, the conversion of pIM cells into MM was achieved, as demonstrated by the presence of SIX2, the MM marker (Figure 1G). In contrast to the normoxic conditions, a notable increase in cells expressing CALB1^+^/SOX9^+^, markers of the UB^[^ ^12^ ^]^ was observed in the hypoxic conditions, accompanied by elevated levels of HIFα (Figure 1G,I). In line with the immunostaining results, the mRNA expression level of *CALB1* was significantly increased while *SIX2* expression was decreased under hypoxia (Figure 1H). This trend was consistently observed across the different batches. We found a significantly higher percentage of SOX9^+^ cells under hypoxia (27.36 ± 0.67%) than under normoxia (1.97 ± 0.86%), whereas the percentage of SIX2^+^ cells was reduced under hypoxia (65.75 ± 1.29%) compared to normoxia (86.71 ± 0.19%) (Figure 1I,J). Localization of CALB1^+^/SOX9^+^ cells around the SIX2^+^ cells resembles the spatial arrangement observed in in vivo development,^[^ ^13^ ^]^ exhibiting consistency with the presence of aIM‐like cells on day 7. There was no overlap between SIX2^+^ MM cells and CALB1^+^/SOX9^+^ UB‐like cells, possibly reflecting the distinct origins of MM and UB.

Continuous treatment with FGF9 from days 9 to 14, together with CHIR from days 9 to 11, successfully induced PAX8^+^ cells on day 14 (Figure 1K), as previously reported.^[^ ^3c^ ^]^ Interestingly, PAX8^+^/SOX9^+^ co‐expressing cells (26.04 ± 1.29%) were prominently observed under hypoxia across various batches, while such cells were hardly detectable under normoxia (3.93 ± 1.10%) (Figure 1K,L). This indicates that both PAX8^+^/SOX9^‐^ RV^[^ ^3c^ ^]^ and PAX8^+^/SOX9^+^ UB‐like cells^[^ ^12b^ ^]^ (Figure 1L) can be spontaneously induced in hypoxic conditions, further demonstrating the sustained presence of SOX9^+^ cells on day 14, even after the withdrawal of hypoxic stimulation.

### 2.2 Characterization of Kidney Organoids Developed under Optimal Hypoxia

**Figure 2:**
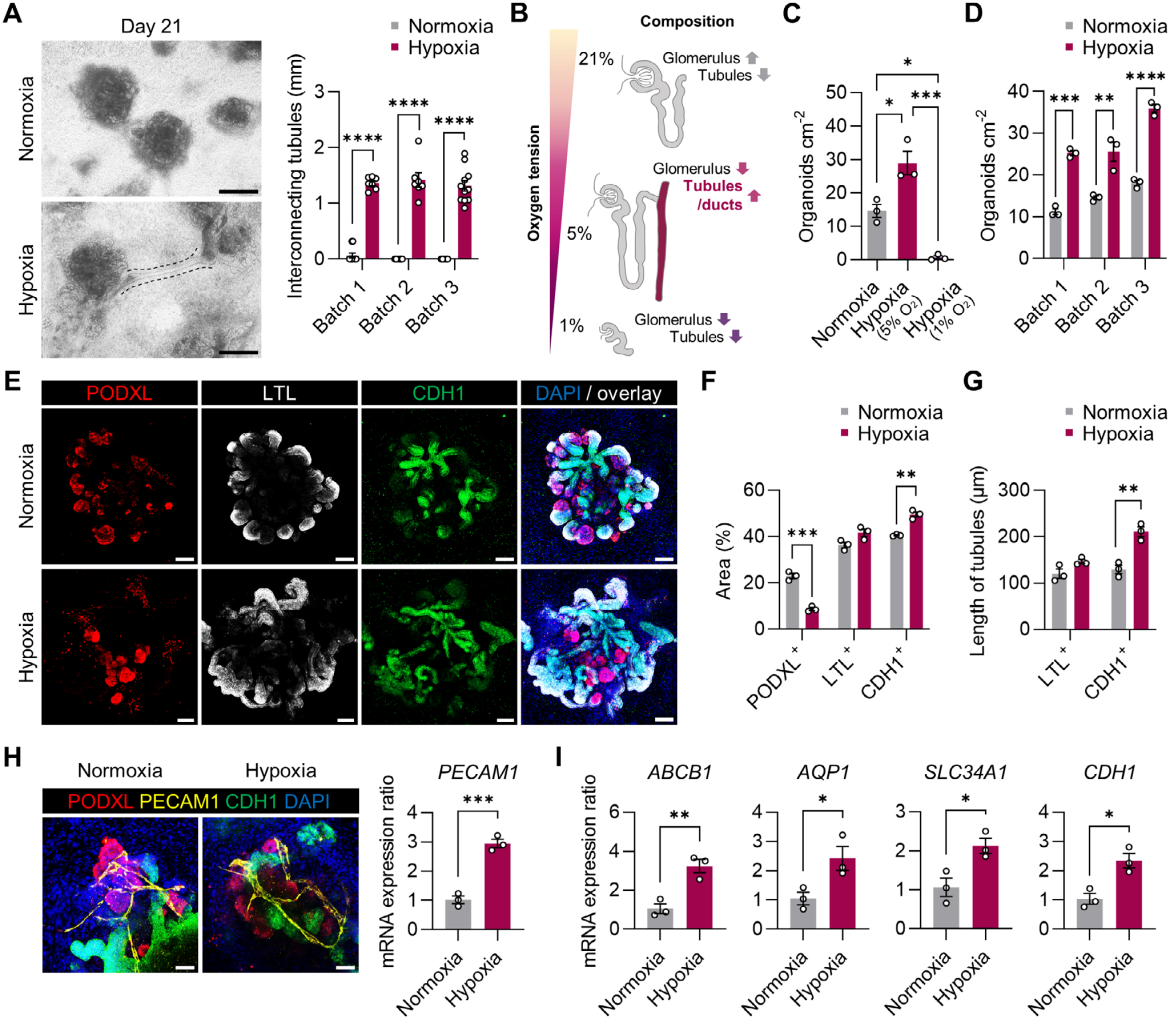
Development of kidney organoids featuring extended and mature tubular structures under the optimized hypoxic conditions. A) Left: Bright‐field images of kidney organoids on day 21 of differentiation in normoxia and hypoxia. Scale bars, 500 µm. Right: The length of the elongated tubules in kidney organoids. B) Diagrams of different compositions of nephron segments in the resulting kidney organoids depending on oxygen tension during the initial nine days. C,D) The number of kidney organoids > 200 µm per cm^2^ under normoxia and hypoxia (5% O_2_ or 1% O_2_) conditions on day 21. E) Fluorescence microscopy images of kidney organoids, stained for a marker of podocytes (PODXL), proximal tubules (LTL), loops of Henle, distal tubules, and collecting ducts (CDH1), on day 21 of differentiation under normoxic and hypoxic conditions. Scale bars, 100 µm. F) The percentage area of kidney organoids positive for PODXL, LTL, or CDH1 on day 21. G) The lengths of LTL^+^ or CDH1^+^ tubules on day 21 of kidney organoids in the normoxic and hypoxic conditions. H) Left: Fluorescence microscopy images of kidney organoid on day 21 stained for PODXL, PECAM1, and CDH1. Scale bars, 100 µm. Right: The ratios of mRNA expression of *PECAM1*, a marker of renal vasculature. I) The ratios of mRNA expression of genes encoding markers for proximal tubules (*ABCB1*, *AQP1*, and *SLC34A1*) and loops of Henle, distal tubules, and collecting ducts (*CDH1*) in the kidney organoids on day 21 differentiated under hypoxia relative to normoxia conditions. All data are plotted as mean ± S.E. and *N* = 3 for the independent experiments. *p* values were determined by (A,D,F,G,H,I) two‐tailed unpaired *t*‐test and (C) one‐way ANOVA followed by Tukey's multiple comparison test (^*^ *p* < 0.05; ^**^ *p* < 0.01; ^***^ *p* < 0.001; ^****^ *p* < 0.0001).

On day 21, we observed that the kidney organoids differentiated in the hypoxic conditions displayed unique tubular structures that interconnected with adjacent nephrons, which resemble the renal morphology where collecting ducts connect to multiple nephrons (**Figure** **2**A,B). The tubules interconnecting nephrons extended to the lengths of 1–2 mm (Figure 2A), a feature not observed in kidney organoids grown without hypoxic stimulation.^[^ ^3c^ ^]^ This suggests that alterations in induction of the progenitors by hypoxic stimulation led to the formation of high‐order kidney organoid structures. Additionally, our analysis revealed that under the optimal hypoxic conditions (5% O_2_), the formation of kidney organoids larger than 200 µm was more efficient compared to those developed under standard oxygen conditions across the different batches (Figure 2C,D).

Confocal microscopic analysis revealed the formation of nephron structures under both normoxic and hypoxic conditions. These structures included PODXL^+^ glomerular podocytes, LTL^+^ proximal tubules, and CDH1^+^/UMOD^+^ intermediate tubules and CDH1^+^/ UMOD^‐^ distal and collecting duct‐like tubules (Figure 2E; Figure S2A, Supporting Information). Under hypoxic conditions, the CDH1^+^ area increased by ≈9% (Normoxia: 40.61 ± 0.23%; Hypoxia: 49.56 ± 1.12%), while the area of PODXL^+^ decreased by ≈14% (Normoxia: 23.01 ± 1.09%; Hypoxia: 8.74 ± 0.65%) (Figure 2F). We observed a notable elongation of CDH1^+^ tubules showing strong expression of OCLN (Figure S2B, Supporting Information), a feature of tight junction in the distal tubules and collecting ducts;^[^ ^14^ ^]^ but no significant change in the area (Normoxia: 36.38 ± 1.24%; Hypoxia: 41.71 ± 1.60%) and length of LTL^+^ proximal tubules (Figure 2F,G). Despite the decreased area of podocytes under the hypoxic conditions, the podocytes exhibited mature characteristics, basal expression of ZO‐1 (tight junction) observed in slit diaphragms (Figure S2C, Supporting Information).^[^ ^15^ ^]^ We also sought to determine whether the optimized hypoxic conditions (5% O_2_ for 9 days) could enhance vascularization in kidney organoids, based on earlier findings that different hypoxic conditions (7% O_2_) at later stages of kidney organoid differentiation increase the endothelial population.^[^ ^16^ ^]^ Although we observed a significant increase in mRNA expression of *PECAM1* in kidney organoids cultured under hypoxic conditions (Figure 2H), there was no noticeable change in renal vascularization. This finding suggests that while the hypoxic stimulation we employed is conducive to the development of renal tubular structures, particularly downstream of the proximal tubules, they may not be optimal for augmenting vascularization in kidney organoids.

Consistent with the enhanced structural features, the qRT‐PCR analysis also showed a significant increase in the expression of functional genes in various renal tubular segments, including proximal tubules (*ABCB1*, *AQP1*, and *SLC34A1*) and loops of Henle (*UMOD*), distal tubules/collecting ducts (*CDH1*) in kidney organoids cultured under hypoxia compared to those under normoxia (Figure 2I; Figure S2D, Supporting Information). Furthermore, statistically significant increases in mRNA expression were observed for genes encoding *NPHS1* ^[^ ^17^ ^]^ (Figure S2D, Supporting Information), involved in glomerular filtration, suggesting that hypoxic stimulation does not hinder glomerular formation but rather promotes the maturation of glomerular cells.^[^ ^18^ ^]^

The gene expression profiles of kidney organoids under hypoxia versus normoxia were further examined by bulk RNA‐sequencing analysis. Among 1124 differentially expressed genes (|log_2_(fold change)| > 1 and *p*‐value < 0.05), 560 genes were up‐regulated and 564 genes were down‐regulated in the kidney organoids cultured under hypoxic conditions compared to those in normoxic conditions (Figure S3A,B, Supporting Information). Of the substantial changes in gene expression owing to the different oxygen levels, *MT1H* and *CALB1*, known to be enriched in mature proximal tubules and collecting ducts, were among the 10 top upregulated genes (Figure S3A, Supporting Information). Conversely, *SIX2* and *EYA1*, which are required for expansion of NPCs, were among the 10 top downregulated genes (Figure S3A, Supporting Information). Consistent with this, mature marker genes of the nephron segments were positively regulated, whereas early nephron and renal progenitor markers were negatively regulated under hypoxia compared to those under normoxia (Figure S3C, Supporting Information). This indicates that mature markers of the renal segments, including the collecting ducts, were positively regulated by hypoxic stimulation.

### 2.3 Examination of Collecting Duct Marker Expression in Hypoxia‐Enhanced Kidney Organoids

**Figure 3:**
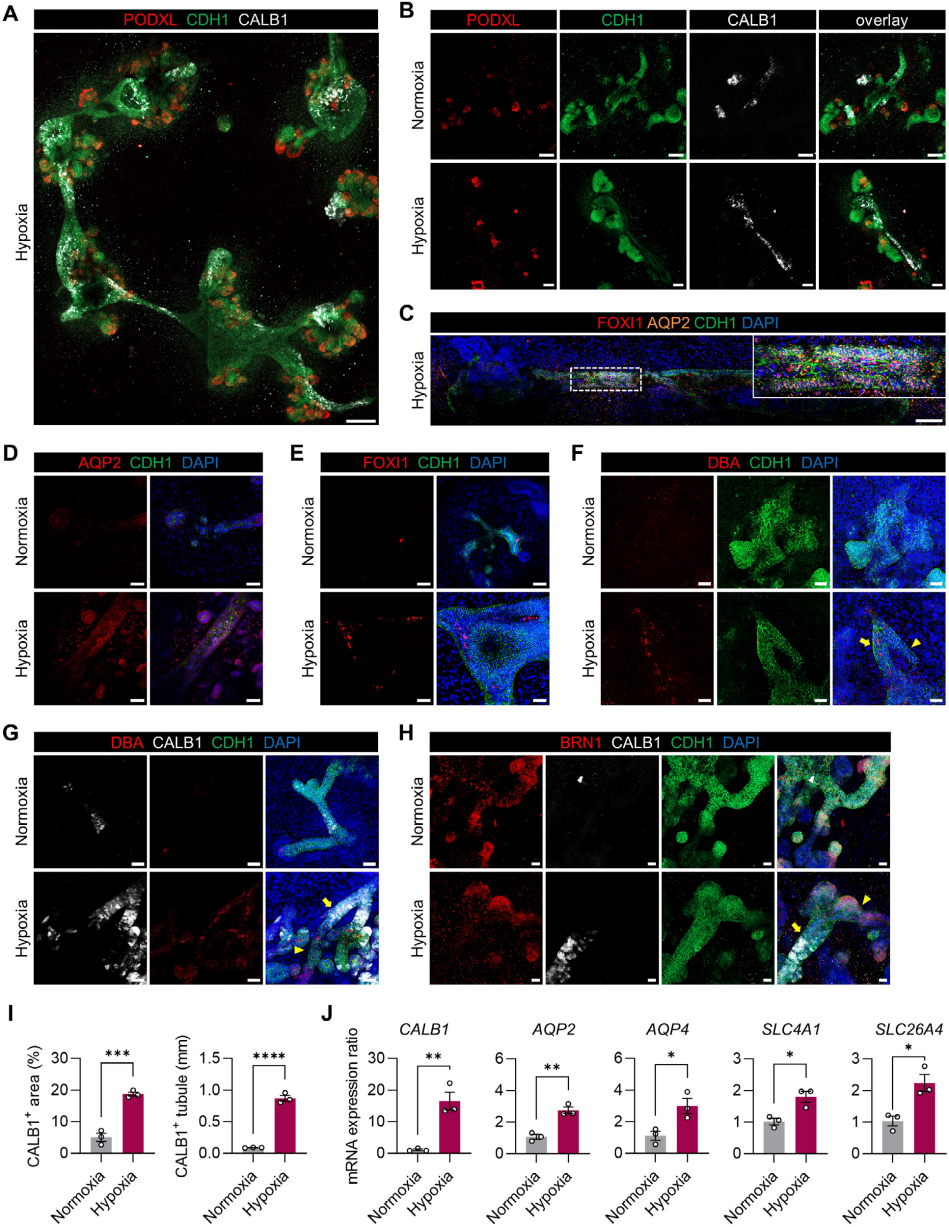
The expression of collecting duct subtype cellular markers in highly structured tubules of hypoxia‐enhanced kidney organoids. A) A fluorescence microscopy image of kidney organoids on day 21, differentiated under hypoxic conditions and stained with podocytes (PODXL), loops of Henle/distal tubules/collecting ducts (CDH1), and collecting ducts (CALB1) markers, showing multiple nephrons connected by a collecting duct tree, resembling the structure of a kidney in vivo. Scale bar, 200 µm. B) Fluorescence microscopy images of kidney organoids on day 21 labeled with PODXL, CDH1, and CALB1, demonstrating higher expression of CALB1 under hypoxia than normoxia. Scale bars, 100 µm. C) Fluorescence microscopy images of hypoxia‐enhanced kidney organoids on day 26 showing intermingled AQP2^+^ and FOXI1^+^ cells within CDH1^+^ tubules. Scale bar, 100 µm. D–H) Immunofluorescence micrographs of the kidney organoids labeled with distal tubules (BRN1 and CDH1) and collecting ducts (CDH1, AQP2, FOXI1, and DBA) markers on day 35 differentiated under normoxia and hypoxia conditions. The arrowheads and arrows indicate the distal tubule and collecting duct‐like segment, respectively. Scale bars, 50 µm (D–G) and 20 µm (H). I) Quantification of CALB1^+^ tubules. Left: The percentage area of CALB1^+^ area relative to CDH1^+^ area. Right: The length of CALB1^+^/CDH1^+^ tubules. J) mRNA expression ratios of genes expressed in principal cells (*AQP2* and *AQP4*), α‐intercalated cells (*SLC4A1*), and β‐intercalated cells (*SLC26A4*). (I,J) All data are plotted as mean ± S.E. and *N* = 3 for the independent experiments. Statistical test: Two‐tailed unpaired *t*‐test. (^*^ *p* < 0.05; ^**^ *p* < 0.01; ^***^ *p* < 0.001; ^****^ *p* < 0.0001).

The substantial changes in the expression level of *CALB1* in hypoxia led us to confirm its expression at the protein level. The confocal microscopic analysis revealed that multiple nephrons (PODXL^+^) were connected via CDH1^+^/CALB1^+^ tubules^[^ ^12a^ ^]^ (**Figure** **3**A), resembling the collecting duct trees typically found in the renal system.^[^ ^19^ ^]^ Both kidney organoids differentiated under normal and hypoxic conditions contained CDH1^+^/CALB1^+^ cells; however, the hypoxia‐enhanced organoids exhibited a greater area and length of continuous CDH1^+^/CALB1^+^ tubules compared to the control organoids (Figure 3B,I).

The functions of collecting duct are carried out by two major cell populations, principal and intercalated cells, which are intermingled throughout the entire network of the collecting ducts. Principal cells concentrate urine and regulate Na^+^/K^+^ homeostasis, whereas intercalated cells regulate normal acid‐base homeostasis via H^+^ or HCO^3−^ in urine.^[^ ^20^ ^]^ When we compared the expression of AQP2 and FOXI1, markers for principal cells and intercalated cells, respectively, we found a notable presence of AQP2^+^ and FOXI1^+^ cells within CDH1^+^ tubular structures in hypoxia‐enhanced kidney organoids compared to normal organoids. (Figure 3C–E). Moreover, there was also enhanced expression of Dolichos biflorus agglutinin (DBA), a highly selective lectin indicating principal cells, in organoids differentiated in hypoxic conditions, in contrast to the normoxia group, where DBA expression was not detected as previously reported^[^ ^3c^ ^]^ (Figure 3F). Co‐expression of DBA and CALB1 was also observed in CDH1⁺ tubules under hypoxia (Figure 3G; Figure S4A, Supporting Information). Additionally, distinct CALB1⁺ and BRN1⁺ distal tubule segments were found within single CDH1⁺ tubules (Figure 3H; Figure S4B, Supporting Information). These results suggest a potential structural connection under hypoxia between the distal tubule segment and the collecting duct‐like segment.^[^ ^3c^ ^]^ Additionally, mRNA expression levels of principal cell marker genes (*AQP2* and *AQP4*) and intercalated cell marker genes (*SLC4A1* and *SLC26A4*) were significantly upregulated in hypoxia‐enhanced organoids (Figure 3J). These findings suggest that hypoxic stimulation can successfully induce the generation of both AQP2^+^, FOXI1^+^, and DBA^+^ cells, while also achieving the structural enhancement of the tubules resembling collecting ducts‐like structures.

The enhancements in tubular structures, including the expression of collecting duct markers, were also validated using another hiPSC line, WTC‐11. The optimal hypoxic conditions effectively promoted the formation of WTC‐11 line‐derived kidney organoids, which exhibited elongated and interconnected tubules across three independent batches (Figure S5A,B, Supporting Information). Alongside the expression of PODXL, LTL, and CDH1, hypoxia‐enhanced kidney organoids showed notable expression of CALB1, DBA, AQP2, and FOXI1 compared to kidney organoids cultured in normoxia (Figure S5C–G, Supporting Information). Together, these data demonstrate a reliable and reproducible generation of kidney organoids showing enhanced tubular structures under our hypoxic conditions across different hiPSC lines and batches.

The beneficial effect of developmentally‐inspired hypoxic conditions on kidney organoid differentiation was evident even during extended culture periods. Specifically, on day 50, the organoids differentiated under hypoxic conditions displayed even more highly branched tubules, resulting in more complex tubular networks than those observed on day 21 (Figure S6A, Supporting Information). This level of complexity was absent in organoids cultured without hypoxic stimulation. While the nephron structures remained well‐preserved under both conditions, the presence of AQP2^+^ and FOXI1^+^ cells was particularly notable in the hypoxia group (Figure S6B–D, Supporting Information). Consistent with previous findings that long‐term culture of kidney organoids results in fibrotic lesions and reduced expression of proliferative capacity,^[^ ^21^ ^]^ we observed PDGFRβ^+^ mesenchymal cells co‐expressing αSMA^+^ (a myofibroblast marker), as well as KI67^+^ cells (a proliferation marker). However, there was no significant difference in αSMA and KI67 expression between the two groups (Figure S6B,E, Supporting Information). Additionally, the elevated expression of mature marker genes, which was evident in the hypoxia‐enhanced kidney organoids on day 21 (Figure 2I; Figure S2D, Supporting Information), continued to be observed up to day 50 (Figure S6F, Supporting Information).

### 2.4 Transcriptomic Analysis of Hypoxia‐Enhanced Kidney Organoids

**Figure 4:**
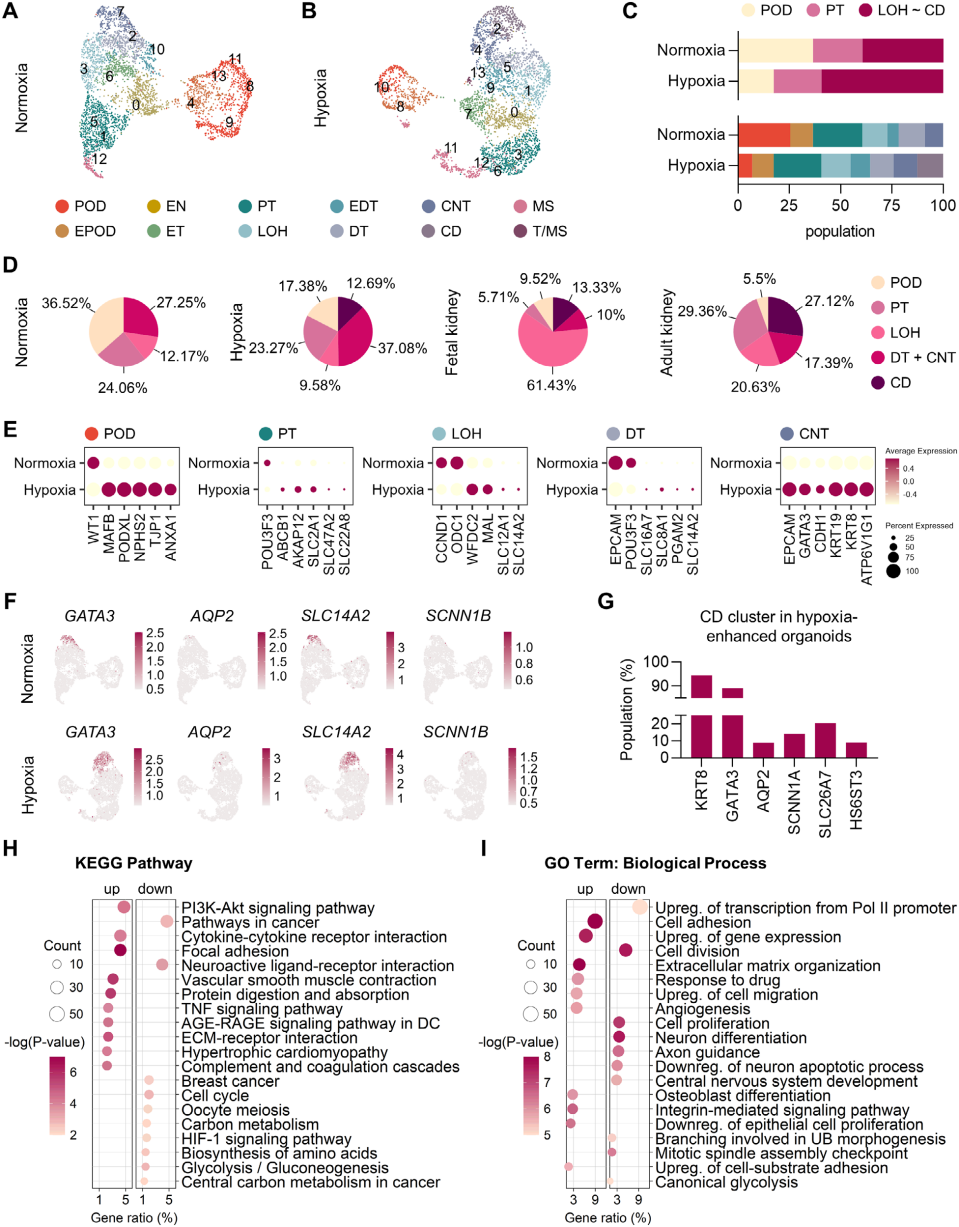
Transcriptomic profiling of kidney organoids showing mature features of kidney organoids. A,B) UMAP plots representing cell type annotated clusters from kidney organoids differentiated under normoxia (A) and hypoxia (B) on day 34. (*N* = 1) C) Bar charts presenting the population of nephron segments (top) and clusters (bottom) in each condition. D) Pie charts showing the ratio of nephron segments in the normoxia, hypoxia, and human fetal^[^ ^37^ ^]^ and adult kidney^[^ ^38^ ^]^ datasets. E) Dot plots showing mRNA expression of key genes in each cell type. F) UMAP plots showing the expression of collecting duct marker genes (*GATA3*, *AQP2*, *SLC14A2*, and *SCNN1B*) in the normoxic and hypoxic conditions. G) Bar charts presenting the percentage of cells expressing principal cell marker genes (*KRT8*, *GATA3*, *AQP2*, and *SCNN1A*) and intercalated cell marker genes (*SLC26A7*, *HS6ST3*) in the CD cluster under hypoxia. H,I) KEGG functional classification (H) and biological process GO term (I) of kidney organoids. The color of dots represents the significance of enrichment, while the size represents the input number of genes for each KEGG pathway and GO terms. The horizontal axis indicates the gene ratio. DC, Diabetic complications; Upreg, Up‐regulation; Pol II, RNA polymerase II; Downreg, Down‐regulation. POD, podocyte; EPOD, early podocyte; EN, early nephron; ET, early tubule; PT, proximal tubule; LOH, loop of Henle; EDT, early distal tubule; DT, distal tubule; CNT, connecting tubule; CD, collecting duct; MS, mesenchyme; T/MS, tubule/mesenchyme.

To compare renal cell types in kidney organoids cultured under hypoxic versus normoxic conditions, single cell RNA‐sequencing was performed on day 34 organoids (**Figure** **4**A,B). A total of 35281 cells were analyzed, with 17163 from normoxia and 18118 from hypoxia, following the previous analytical strategy.^[^ ^22^ ^]^ Unsupervised clustering revealed 17 clusters in normoxic organoids and 16 in hypoxic ones, with both groups containing typical kidney cell types, such as podocytes, tubules, and mesenchyme, as well as off‐target cells (e.g., neuron, muscle, and proliferating cells), consistent with the previous reports.^[^ ^22^ ^]^ The proportion of differentiated kidney cells (podocytes, tubules, and mesenchyme) was comparable between the two conditions: 78.14% in hypoxia and 75.81% in normoxia, with no significant differences in off‐target cells (Figure S7A–D and Table S1, Supporting Information).

Five clusters from each condition, identified by *MAFB* or *EPCAM* expression as podocytes or tubular epithelial cells,^[^ ^23^ ^]^ were further sub‐clustered to examine renal cells in greater detail (Figure S7A–D, Supporting Information). Fourteen distinct clusters were identified in both conditions (Figure 4A,B; Figure S7E,F, Supporting Information). Notably, a cluster expressing *SOX5* and *GPX2*, marker genes detected in the human collecting duct,^[^ ^24^ ^]^ was only found in hypoxia‐enhanced organoids (Figure 4A–C; Figure S7E,F, Supporting Information). Consistent with immunofluorescent analysis, the proportion of podocytes decreased by 19.14% in hypoxia (Figure 4C; Table S2, Supporting Information), while the population of remaining tubular cells, excluding proximal tubular cells, increased by 19.92% (Figure 4C). When we compared the cellular composition of organoids relative to fetal and adult kidney datasets using the previous approach,^[^ ^25^ ^]^ we observed a closer resemblance between hypoxia‐enhanced kidney organoids and the human adult kidney than that of the fetal kidney, in contrast to the normoxia group (Figure 4D).

Kidney organoids under hypoxia exhibited increased expression of the mature marker genes, each analyzed specifically within its corresponding cell type clusters: podocyte (POD; *NPHS2*, *TJP1*,^[^ ^26^ ^]^ and *ANXA1* ^[^ ^27^ ^]^), proximal tubule (PT; *SLC47A2* and *SLC22A8*), loops of Henle (LOH; *WFDC2* and *SLC12A1*), distal tubule (DT; *SLC14A2* and *SLC8A1*), and connecting tubule (CNT; *KRT19* and *ATP6V1G1*) (Figure 4E).^[^ ^22^, ^28^ ^]^ Although confocal microscopy observed the notable expression of AQP2^+^ and FOXI1^+^ cells, only a small population of cells expressed key functional marker genes for collecting ducts,^[^ ^29^ ^]^ including *AQP2*, *SLC14A2*, and *SCNN1B*, under hypoxia (Figure 4F). Under hypoxia, we analyzed the expression of marker genes for principal cells (*KRT8*, *GATA3*, *AQP2*, *SCNN1A*) and intercalated cells (*SLC26A7*, *HS6ST3*) specifically within the collecting duct‐like cluster (CD). Most cells expressed *KRT8* and *GATA3*, which are also found in the UB in addition to the collecting duct,^[^ ^30^ ^]^ while only a small fraction expressed the mature collecting duct markers *AQP2*, *SCNN1A*, *SLC26A7*, and *HS6ST3* (Figure 4G). This suggests that while hypoxia can promote the generation of collecting duct‐like cells, their level of maturity remains low. Furthermore, the low proportion of intercalated cell marker expression suggests the need for protocol refinement, as healthy kidney tissue typically has a higher proportion of intercalated cells, comprising ≈30% of collecting duct cells.^[^ ^31^ ^]^

We additionally performed reference‐based mapping using a healthy adult human kidney dataset from the Kidney Precision Medicine Project (KPMP)^[^ ^24^ ^]^ to provide external support for the identity of collecting duct‐like cells (Figure S8A,B, Supporting Information). This analysis revealed a consistent increase in collecting duct‐like cells under hypoxic conditions compared to normoxia (Figure S8C, Supporting Information). The prediction scores for these cells (Figure S8D,E, Supporting Information) were similar to those reported in a previous study mapping human UB organoids to adult tissue references.^[^ ^30^ ^]^ The modest scores are likely attributable to the immaturity of organoid‐derived cells compared to adult kidney tissue^[^ ^30^ ^]^ and the small number of cells (fewer than 100 per cluster). Notably, collecting duct‐like cells showed a trend toward higher prediction scores under hypoxia compared to normoxia, suggesting improved lineage specification, though further studies will be required to enhance organoid maturation.

To identify pathways and biological processes involved in kidney development under hypoxia, KEGG pathway and Gene Ontology (GO) enrichment analyses were performed based on bulk RNA‐seq. The PI3K–Akt signaling pathway, crucial for UB branching,^[^ ^32^ ^]^ was the most enriched pathway (Figure 4H). Additionally, genes related to focal adhesion, including *ACTN1*, *COL1A1*, *COL6A6*, and *FN1*, were significantly upregulated, corresponding with the development of complex tubular structures^[^ ^33^ ^]^ observed in hypoxia‐enhanced organoids (Figure 4H).

The most enriched biological process GO term was cell adhesion and extracellular matrix (ECM) organization (Figure 4I). Genes involved in cell adhesion, critical in the kidney tubular function and morphogenesis,^[^ ^34^ ^]^ such as *CLDN2* and *CLDN11*, were upregulated in hypoxia‐enhanced organoids. These genes encode tight junction proteins that regulate paracellular transport in kidney tubules, aligning with the improved tubular structures observed.^[^ ^35^ ^]^ Furthermore, genes associated with cell division and positive regulation of transcription from RNA polymerase II promoters were downregulated (Figure 4I), which aligns with the reduced oncogenic potential suggested by the KEGG pathway analysis (Figure 4H). In terms of molecular function, genes related to calcium binding, such as *RYR2*, *CALB1*, *ANXA1*, and *SPARC*, were upregulated in hypoxia‐enhanced organoids (Figure S3D, Supporting Information). This indicates that hypoxia may activate calcium signaling, which plays a crucial role in converting UB and MM into functional nephron epithelium during kidney development.^[^ ^36^ ^]^

### 2.5 Tubular Microstructure and Functionality of Hypoxia‐Enhanced Kidney Organoids

**Figure 5:**
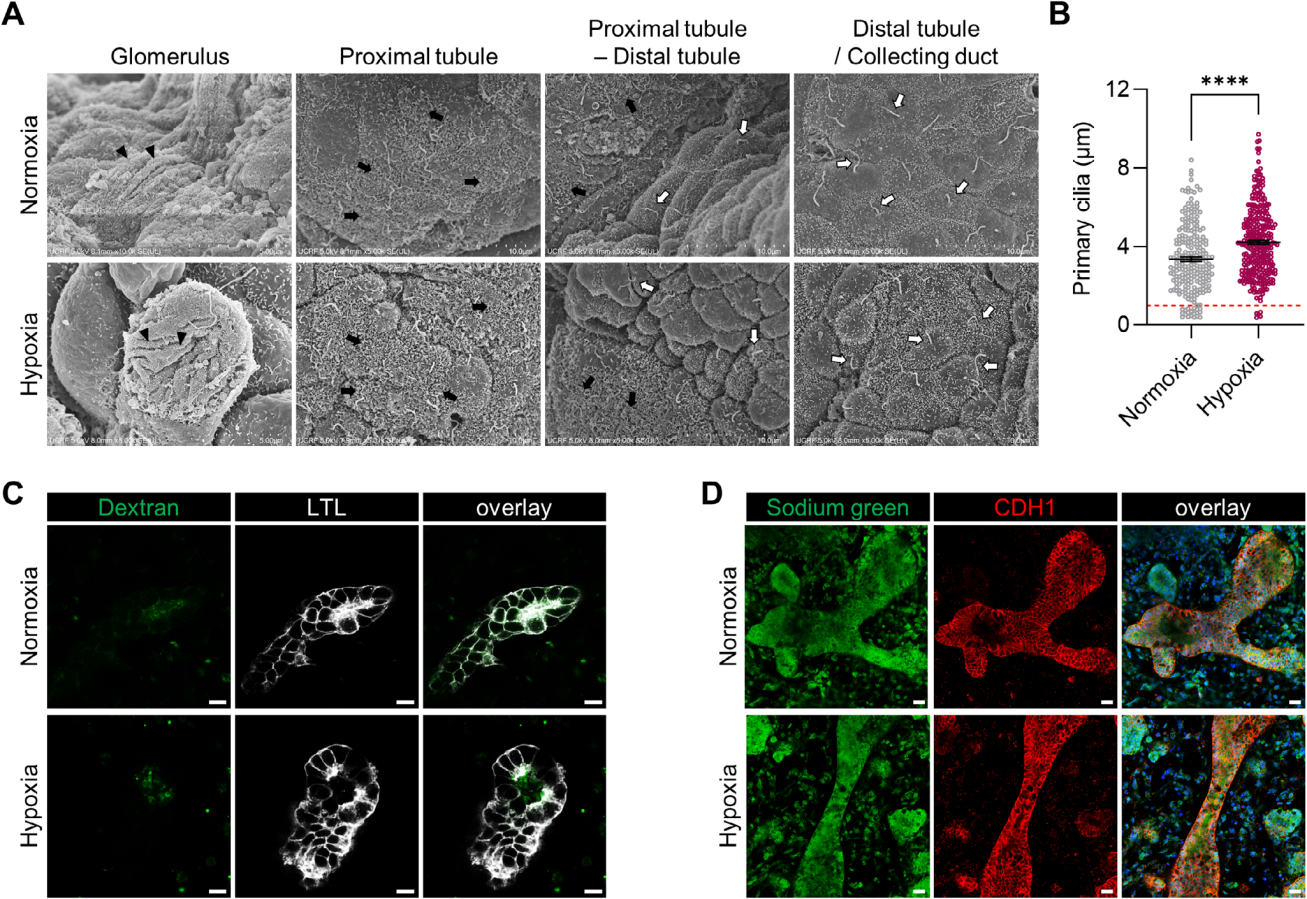
Nephron segments displaying mature microstructural characteristics and functional tubular elements. A) Electron micrographs showing each nephron segment of human kidney organoids differentiated in normoxia and hypoxia conditions. The arrowheads, black arrows, and white arrows indicate podocyte projections, brush border microvilli, and primary cilia, respectively. B) The length of primary cilia in kidney organoids on day 21, analyzed by ImageJ. All data are plotted as mean ± S.E. and *N* = 3 for the independent experiments. Statistical analysis is a two‐tailed unpaired *t*‐test (^****^ *p* < 0.0001). C) Dextran uptake assay showing endocytic uptake of LTL^+^ proximal tubules. Scale bars, 10 µm. D) Sodium uptake assay showing sodium green absorption of CDH1^+^ tubules. Scale bars, 20 µm.

The development of cilia in nephrons has been linked to kidney development, with average length of cilia in developing kidney being < 1 µm and 3–10 µm in quiescent and fully differentiated cells.^[^ ^39^ ^]^ Electron microscopic analysis revealed that a significantly lower percentage of cells in the hypoxia‐enhanced organoids (1.66%) had primary cilia in the distal tubules and collecting duct‐like tubules that were shorter than 1 µm compared to those of the normal kidney organoids (8.37%) (**Figure** **5**A,B). This indicates that the hypoxia‐enhanced kidney organoids contained relatively few immature cells. In addition, 72.19% cilia with lengths between 3 and 10 µm were observed in hypoxia‐enhanced kidney organoids, which was 20.2% higher than those in the normal kidney organoids (Figure 5B).

Furthermore, the proximal tubules had a well‐developed brush border that increases their apical surface area (Figure 5A). We observed denser and longer microvilli in the proximal tubules of hypoxia‐enhanced kidney organoids than in normal kidney organoids (Figure 5A). Distinctive short and sparse microvilli were also observed in the distal tubules and collecting duct‐like tubules of hypoxia‐enhanced organoids, which were similar to those of mammalian renal tissue,^[^ ^40^ ^]^ whereas the distal and collecting duct‐like tubules in the normoxic kidney organoids exhibited relatively flatter structures owing to less developed microvilli (Figure 5A). Both organoids exhibited glomerular podocyte‐like structures with numerous projections (Figure 5A). These findings suggest that developmentally‐inspired hypoxic stimuli supported the anatomical maturation of tubular epithelia in kidney organoids.

To examine whether hypoxia‐enhanced kidney organoids exhibit physiologically relevant functions, we conducted an in vitro dextran uptake assay based on previously established methods.^[^ ^3a,e^ ^]^ The organoids were exposed to 10 kDa dextran for 24 h, and we observed that they selectively absorbed and retained dextran in LTL^+^ proximal tubules (Figure 5C), suggesting their capability for reabsorption. After confirming functionality of proximal tubules in hypoxia, we performed an in vitro sodium uptake assay to evaluate functionality of CDH1^+^ tubules.^[^ ^41^ ^]^ To prevent efflux of sodium ion through basolateral Na^+^/K^+^‐ATPase, cells were incubated with ouabain for 24 h and then treated with sodium green for 3 h. We observed intracellular sodium green within CDH1^+^ tubules (Figure 5D), confirming the functionality of the distal and collecting duct‐like tubules in the hypoxia‐conditioned kidney organoids.

### 2.6 The Effect of Hypoxia on Reciprocal Signaling Genes Involved in the Development of the MM and UB

**Figure 6:**
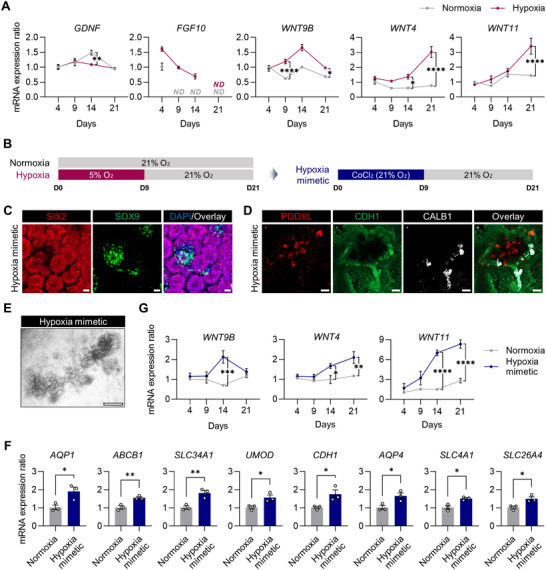
Improved tubular development by HIF1α‐induced commutual inductive signals. A) mRNA expression ratios of genes encoding *GDNF*, *FGF10*, *WNT9B*, *WNT4*, and *WNT11* analyzed over time and compared to their expression on day 4 under hypoxic versus normoxic conditions. *ND*, non‐detected. B) Timeline for in vitro differentiation of kidney organoids using CoCl_2_ as a hypoxia‐mimetic agent. C,D) Immunofluorescence micrographs of kidney organoids treated with CoCl_2_ for an initial nine days, labeled with antibodies against SIX2 and SOX9 on day 9 (C), and PODXL, CDH1, and CALB1 on day 21 (D). Scale bars, 20 µm (C) and 100 µm (D). E) Bright‐field images of CoCl_2_‐treated kidney organoids on day 21. Scale bar, 500 µm. F) The mRNA expression ratios of genes encoding markers for proximal tubules (*AQP1*, *ABCB1*, and *SLC34A1*), loops of Henle (*UMOD* and *CDH1*), distal tubules (*CDH1*) and collecting ducts (*CDH1*, *AQP4*, *SLC4A1*, and *SLC26A4*) in the kidney organoids on day 21 differentiated under the hypoxia relative to normoxia conditions. G) mRNA expression ratios of *WNT9B*, *WNT4*, and *WNT11*, compared to their expression on day 4 under hypoxic versus normoxic conditions. (A,F,G) All data are plotted as mean ± S.E. and *N* = 3 for the independent experiments. *P* values were determined by (A,G) two‐way ANOVA followed by Šídák's multiple comparison test and (F) two‐tailed unpaired *t*‐test (^*^ *p* < 0.05; ^**^ *p* < 0.01; ^***^ *p* < 0.001; ^****^ *p* < 0.0001).

To comprehend how hypoxic stimuli during differentiation lead to the generation of kidney organoids with higher‐order structures and improved maturity, we investigated whether there are changes in the expression levels of genes involved in reciprocal signaling between the MM and UB, which are essential for forming complex kidney structures in vivo.^[^ ^2^ ^]^ UB stimulates MM to differentiate into glomeruli and renal tubular epithelia, and in turn, MM furthers the branching of UB and helps it differentiate into the collecting ducts. In mice, GDNF is regarded as a critical factor for UB outgrowth from the IM through its receptor Ret,^[^ ^42^ ^]^ and FGF10 collaborates with GDNF to encourage UB growth.^[^ ^43^ ^]^ Unexpectedly, we did not find significant upregulation of *GDNF* mRNA levels in the hypoxia group (**Figure** **6**A). As *GDNF* is activated by *SIX2*,^[^ ^44^ ^]^ the decreased expression of *SIX2* on day 9 may lead to reduced *GDNF* expression on day 14 (Figure 1I and 6A). In contrast, we detected *FGF10* mRNA expression only in the hypoxia group (Figure 6A), suggesting that increased *FGF10* expression has played a role in the development of hypoxia‐enhanced kidney organoids.^[^ ^43^ ^]^ In addition to the compensatory function of FGF in UB morphogenesis, previous observations of UB formation in GDNF knockout mice prompted us to investigate the contribution of the AKT pathway, proposed as the common pathway promoting UB formation whether the GDNF is present or absent.^[^ ^45^ ^]^ We found that *MDM2*,^[^ ^46^ ^]^ *NOTCH1*,^[^ ^47^ ^]^ and *PTEN*,^[^ ^48^ ^]^ the AKT regulatory transcriptional factors known to be involved in UB development, were regulated to activate AKT under hypoxia on day 9 (Figure S9A–C, Supporting Information). The consistency in the enriched PI3K–AKT signaling pathway (Figure 4H) supports the involvement of AKT pathway in organoid development under hypoxia, signifying that GDNF may not be the key factor regulating enhanced organogenesis in hypoxic conditions.^[^ ^45^ ^]^ In response, UB secretes WNT9B that activates WNT4 and promotes the differentiation of some NPCs into nephron components.^[^ ^49^ ^]^ We discovered that *WNT9B* mRNA levels increased by the hypoxia‐stimulation protocol (Figure 6A), and accordingly, the significantly upregulated *WNT4* is similar to the in vivo developmental process (Figure 6A).^[^ ^49c^ ^]^ WNT11 in UB tips creates a positive feedback loop in the GDNF/Ret pathway, thereby contributing to the formation of the collecting duct system.^[^ ^50^ ^]^ Compared to the normoxia group, *WNT11* mRNA expression was significantly elevated on day 21 in the hypoxia group (Figure 6A), indicating an improved signal known to be necessary for the development and maturation of collecting ducts.

Previous studies have indicated that the Wnt/β‐catenin signaling pathway interacts with HIF1α signaling during development.^[^ ^51^ ^]^ We noticed increased levels of HIF1α under hypoxic conditions using western blot and confocal immunofluorescent microscopic analyses (Figure 1B,G). To determine whether HIF1α altered the expression of WNT genes, we treated hiPSCs with 100 µm CoCl_2_ under normal oxygen levels for the initial nine days (Figure 6B); CoCl_2_ imitates hypoxia by stabilizing HIF1α.^[^ ^52^ ^]^ Similar to the hypoxic conditions, SOX9⁺ cell clusters were consistently found interspersed within clusters of SIX2⁺ cells on day 9 (Figure 6C; Figure S10, Supporting Information). By day 21, both CoCl_2_‐treated and hypoxia‐stimulated kidney organoids exhibited similar structures, with multiple nephrons arranged around the CDH1^+^/CALB1^+^ tree, and showed increased expression of functional renal genes compared to those under normoxic conditions (Figure 6D–F). Interestingly, the expressions of *WNT4*, *WNT9B*, and *WNT11* were upregulated in CoCl_2_‐treated kidney organoids (Figure 6G), exhibiting patterns similar to those observed in the hypoxia‐stimulated kidney organoids. These findings suggest that HIF1α accumulation upregulates WNT signaling by supporting the development of UB‐derived cells and promoting reciprocal signaling, ultimately enhancing tubular development and maturation, as observed during normal kidney development in animals.

### 2.7 Hypoxia‐Enhanced Kidney Organoids as Renal Cystic Models for Effective Drug Screening and Injury Models for Toxicity Testing

PKDs are a group of inherited disorders that cause the formation of bilateral cysts in the kidneys.^[^ ^53^ ^]^ Studies on human cyst epithelial cells and PKD animal models have highlighted the crucial role of cAMP in the development of PKD. A decrease in intracellular Ca^2+^ level triggers a change in the cellular response to cAMP, and the activation of extracellular signal‐regulated kinase by cAMP leads to cyst growth.^[^ ^54^ ^]^ Additionally, cAMP stimulates the secretion of chloride and fluid through chloride channels located in the proximal tubules and collecting ducts, including the cystic fibrosis transmembrane conductance regulator (CFTR) and calcium‐dependent chloride channel, anoctamin1 (ANO1).^[^ ^55^ ^]^ Therefore, PKD treatment currently focuses on reducing renal cAMP levels, increasing Ca^2+^, and inhibiting CFTR/ANO1 to decrease cyst growth.^[^ ^56^ ^]^

**Figure 7:**
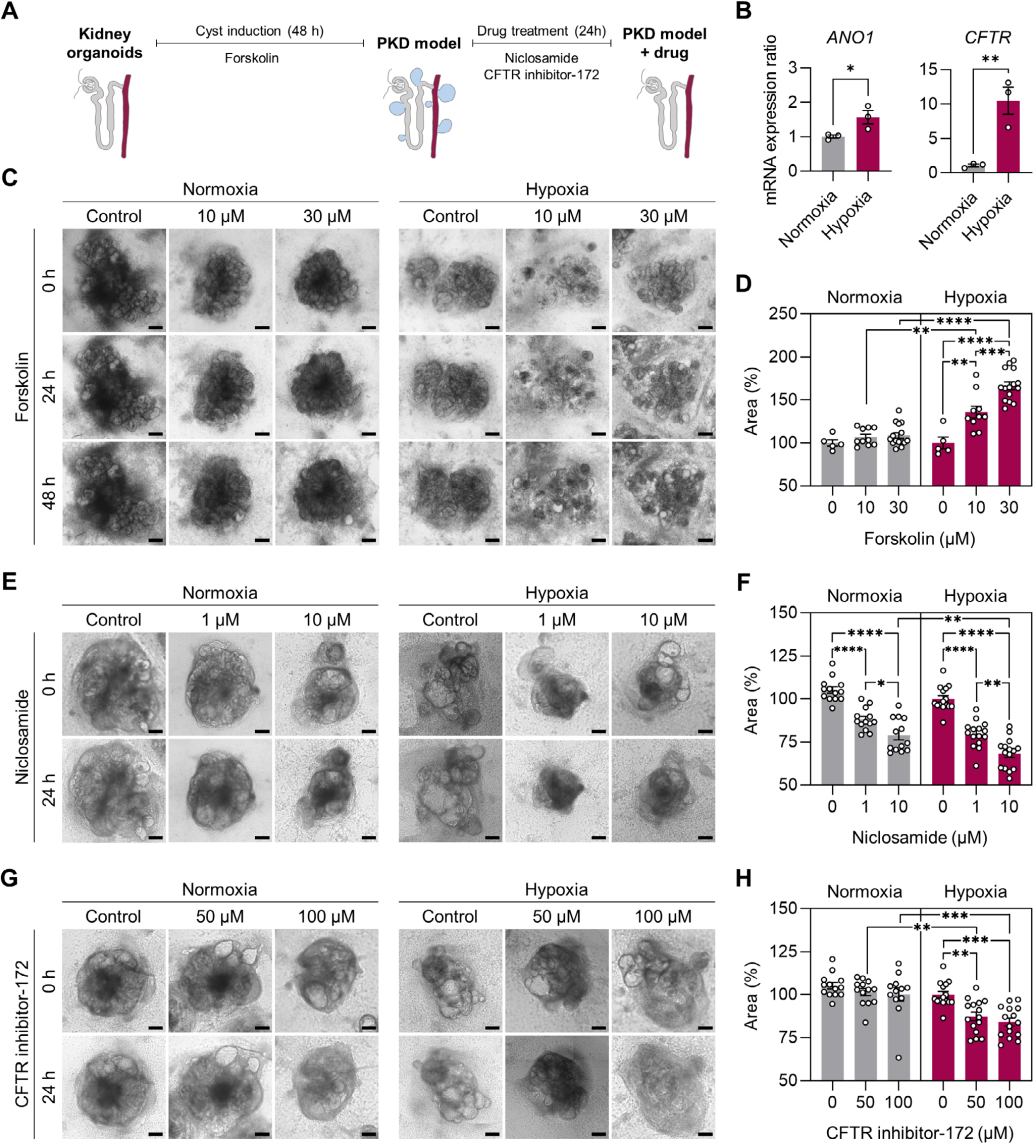
Modeling renal cyst formation with hypoxia‐enhanced kidney organoids for reliable screening of PKD drugs. A) Schematic of the timeline for cyst induction and drug treatment of cultured kidney organoids under normoxia and hypoxia. B) Relative mRNA expression of genes encoding chloride channels (*CFTR* and *ANO1*) in kidney organoids differentiated under hypoxia and normoxia. *p* values were determined by two‐tailed unpaired *t*‐test (^*^ *p* < 0.05; ^**^ *p* < 0.01). C) Bright‐field images of forskolin‐treated kidney organoids differentiated under normoxic and hypoxic conditions. Scale bars, 200 µm. D) The percentage area of organoids treated with forskolin compared to that before induction of cystogenesis. E,G) Bright‐field images of cystic kidney organoids treated with chloride channel inhibitors, niclosamine (E) and CFTR inhibitor‐172 (CFTRinh‐172) (G) for 24 h. The cystic kidney organoids were generated by treating organoids with forskolin 30 µm for 48 h. Scale bars, 200 µm. F,H) The percentage area of kidney organoids after treatment with niclosamine (F) or CFTRinh‐172 (H) for 24 h compared to that of the non‐treated group. All data are plotted as mean ± S.E. and *N* = 3 for the independent experiments. (D,F,H) Statistical analysis is two‐way ANOVA followed by Tukey's multiple comparison test (^*^ *p* < 0.05; ^**^ *p* < 0.01; ^***^ *p* < 0.001; ^****^ *p* < 0.0001).

We found that the mRNA levels of *CFTR* and *ANO1* were significantly increased in kidney organoids subjected to hypoxic stimulation, inducing the formation of well‐structured tubules (**Figure** **7**B). Based on this finding, we hypothesized that renal cystic tissues could be more effectively modeled by treating hypoxia‐stimulated organoids with forskolin, which is used to increase cAMP levels in PKD models.^[^ ^3^, ^6^, ^57^ ^]^ To test this hypothesis, we monitored cystogenesis in kidney organoids after exposure to forskolin (10 and 30 µm) on day 21 (Figure 7A). Remarkably, multiple renal cysts formed 24 and 48 h after exposure to forskolin in hypoxia‐enhanced kidney organoids, whereas normoxic organoids showed less distinct cystogenesis under the same forskolin treatment conditions (Figure 7C). Histological section and confocal immunofluorescence confirmed cyst formation in the inner parts of organoids differentiated in both conditions (Figure S11A,B, Supporting Information). Highly enlarged cysts were robustly formed in hypoxia‐enhanced kidney organoids (Figure S11A, Supporting Information), particularly striking in CDH1^+^ tubules (Figure S11B, Supporting Information). We further observed a significant dose‐dependent increase in the organoids area, resulting from cyst formation by the hypoxia protocol (Figure 7C,D). Additionally, the volume of individual cysts and the number of cysts per unit area were significantly higher in hypoxic than in normoxic kidney organoids after exposure to 30 µm forskolin for 48 h (Figure S11C,D, Supporting Information). In patients with PKD, cAMP activates AQP2, resulting in enhanced water permeability of the collecting ducts.^[^ ^58^ ^]^ Similarly, we found that mRNA expression of *AQP2* significantly increased when hypoxia‐enhanced kidney organoids were exposed to 30 µm forskolin (Figure S11E, Supporting Information). These results suggest that the hypoxia‐enhanced organoids can efficiently mimic cAMP‐mediated cyst formation, even without genetic mutations in *PKD1* or *PKD2*, likely because of robust formation of renal tubules with well‐developed ion channels and transporters.

To evaluate the potential of our kidney model as a drug‐screening tool for PKDs, we tested the therapeutic effects of inhibiting ANO1 and CFTR in the renal cystic model. The ANO1 inhibitor, niclosamide, approved by the Food and Drug Administration, effectively suppressed cyst growth by blocking Cl^−^ current and inhibiting ANO1 expression.^[^ ^59^ ^]^ Cyst‐induced kidney organoids treated with forskolin for 48 h were treated with niclosamide for an additional 24 h, and cyst growth was monitored by microscopy (Figure 7A). Both normoxia and hypoxia‐stimulated kidney organoids showed a significant decrease in organoid size when treated with 1 and 10 µM niclosamide (Figure 7E,F). CFTR inhibitor‐172 (CFTRinh‐172), an allosteric inhibitor that targets the cytoplasmic face of CFTR, slows cyst growth in PKD mice and in vitro models.^[^ ^59^, ^60^ ^]^ Interestingly, a significant decrease in the size of kidney organoids was observed with CFTRinh‐172 at 50 and 100 µm, particularly in hypoxia‐stimulated organoids with relatively high *CFTR* mRNA expression, but not in the normoxic kidney organoids (Figure 7G,H). These results demonstrate that the proposed organoids can efficiently reproduce the therapeutic effects of PKD drugs by reducing cysts.

Next, we further explored the ability of our hypoxia‐enhanced kidney organoids to replicate drug‐induced nephrotoxicity. Previous studies have reported a selective increase in kidney injury molecule‐1 (KIM‐1) expression in the proximal tubules of kidney organoids in response to cisplatin, a nephrotoxic chemotherapeutic agent.^[^ ^3^, ^61^ ^]^ Following the previous findings, we exposed kidney organoids to cisplatin (5 and 50 µm) for 24 h (Figure S12A, Supporting Information). We also observed co‐localized expression of KIM‐1 within the LTL^+^ proximal tubules under both conditions (Figure S12B, Supporting Information). Notably, the percentage of the KIM‐1^+^ area relative to the LTL^+^ area was significantly increased under hypoxia following cisplatin treatment (Figure S12C, Supporting Information). In addition to the quantification results, we observed a notable increase in the mRNA level of *HAVCR1*, the gene encoding KIM‐1, when organoids were exposed to 5 µm cisplatin (Figure S12D, Supporting Information), demonstrating the heightened susceptibility of our hypoxia‐enhanced kidney organoids to cisplatin‐induced nephrotoxicity. This result correlates with the increased expression of *SLC22A8* ^[^ ^28^ ^]^ (Figure 4E), further highlighting the matured characteristics of hypoxia‐enhanced kidney organoids. Collectively, these findings highlight the potential of hypoxia‐enhanced kidney organoids as promising in vitro tools for modeling human pathologies and evaluating drug efficacy and toxicity.

## 3 Discussion and Conclusion

This study demonstrates that applying hypoxic conditions inspired by renal development^[^ ^10b^ ^]^ significantly improves kidney organoid formation, resulting in better tubular structures and maturation. Hypoxia induces the formation of multiple MM‐derived nephrons, connected by UB‐derived collecting duct‐like tubules, more closely resembling physiological kidney structures than previous models.^[^ ^3^ ^]^ Notably, the hypoxia‐enhanced kidney organoids exhibited increased expression of collecting duct markers within their elongated tubules. These organoids also showed greater maturity, characterized by longer primary cilia and higher expression of functional markers for each nephron segment, suggesting that hypoxic stimuli enhance the developmental program of kidney organoids by regulating commitment of progenitors. Unlike previous approaches that separately combined MM‐ and UB‐derived organoids, this hypoxic protocol is simpler and may contribute to the generation of both progenitor cell types within the same culture dish while preserving vascular structures.

Precise control of oxygen levels and exposure duration was crucial, as continuous hypoxia or excessively low oxygen levels failed to produce well‐structured organoids. We hypothesize that biological responses to reoxygenation after 9 days of hypoxia could play an important role, warranting further investigation. WNT signaling, essential for MM‐UB interactions in kidney formation, was significantly upregulated under optimal hypoxic conditions. We also confirmed the involvement of HIF1α‐mediated pathways, key in kidney development.^[^ ^10b^ ^]^ This HIF1α driven upregulation of inductive signals, such as *WNT* and *FGF10*, resulted in maturation of hypoxia‐enhanced kidney organoids compared to normal kidney organoids. Further transcriptomic profiling of the progenitor stages will be informative for understanding the comprehensive mechanisms underlying the development of hypoxia‐enhanced kidney organoids at early stages and upon reoxygenation. Our hypoxic approach can be adapted to other kidney organoid protocols^[^ ^3^, ^5^ ^]^ by fine‐tuning oxygen tension according to the specific strategy.

Importantly, hypoxia‐enhanced organoids hold promise for modeling renal cystic diseases and injury. Unlike prior PKD organoid models,^[^ ^6c^ ^]^ the hypoxia‐enhanced model generates cysts throughout the tubules, including the distal and collecting‐duct like tubules, closely mimicking in vivo conditions.^[^ ^62^ ^]^ This efficient cyst modeling, further improved by chemical induction with forskolin, is attributed to features such as longer primary cilia and increased expression of ANO1 and CFTR. Given the role of primary cilia in sensing mechanical stimuli, integrating hypoxia‐enhanced organoids with microfluidic platforms and PKD mutations could uncover how genetic defects in mechano‐transduction accelerate cyst formation in PKD.^[^ ^63^ ^]^ Moreover, the matured hypoxia‐enhanced kidney organoids exhibited increased expression of transporter systems, which rendered them particularly vulnerable to cisplatin. Profiling transcriptional changes could be leveraged to discover novel biomarkers and therapeutic candidates for acute kidney injury.^[^ ^61^ ^]^ The enhanced susceptibility to nephrotoxins and robust cyst induction demonstrate that our model potentially serves as a more comprehensive tool for preclinical drug development and the identification of treatments for renal injuries and diseases that affect the entire kidney structure.

Despite these advances, further optimization and characterization are necessary. In contrast to the well‐structured tubules expressing collecting duct markers observed in immunofluorescence images, single‐cell sequencing analysis revealed only a very small proportion of cells expressing mature collecting duct marker genes. Further refinement of the hypoxia‐based differentiation protocol is necessary to improve maturation. Spatial mapping of collecting duct‐like cells could provide deeper insights into their distribution within the elongated tubular structures of hypoxia‐enhanced kidney organoids, enabling more precise characterization. In addition, the organoids lacked ureter connections to collecting ducts, essential for filtrate removal, indicating a limitation in fully replicating kidney functionality. Advanced techniques like 3D bioprinting and micro‐physiological systems could help recreate more complex kidney structures, including open nephric ducts. Improving vascularization and optimizing oxygen conditions for vasculogenesis and tubulogenesis^[^ ^16^ ^]^ would further enhance organoid complexity. Additionally, further maturation through chemical and mechanical stimuli and more detailed evaluation of collecting duct cells are needed for applications in tissue engineering.

In summary, our study demonstrated that kidney organoids exposed to developmentally‐inspired hypoxic differentiation conditions exhibited enhanced complexity and maturity compared to those of previous kidney models. Enhanced reciprocal signaling genes, essential for the development of the MM and UB, led to improved tubular structures, maturation of kidney organoids, longer primary cilia, and higher expression of marker genes and proteins in each tubular segment. This advancement further improved the efficiency of modeling renal cysts within the entire kidney structure and enhanced drug testing capabilities. Therefore, these organoids may be useful for modeling physiology and pathophysiology throughout the entire course of branching morphogenesis of the kidney and for drug screening by efficiently recapitulating the in vivo environment of the kidney.

## 4 Experimental Section

### hiPSCs Maintenance

IMR90‐4 and WTC‐11 human induced pluripotent stem cells purchased from WiCell Research Institute and Coriell Institute for Medical Research, respectively. hiPSCs were maintained in mTeSR1 (Stem Cell Technologies, 85850) in 6‐well cell culture plates (SPL Life Sciences, 30006) coated with 1% (v/v) LDEV‐free hESC‐qualified Geltrex (Thermo Fisher Scientific, A1413302) in an incubator at 37 °C with 5% CO_2_. hiPSCs were passaged using Versene (Thermo Fisher Scientific, 15040066) at 1:4 to 1:6 ratio every three–four days according to the manufacturer's protocols.

### Differentiation of hiPSCs

Kidney organoids were generated using a previously published protocol^[^ ^3c^ ^]^ with modification of the O_2_ conditions. hiPSCs were dissociated using Accutase (Sigma–Aldrich, A6964) and then seeded at a density of 3.8 × 10⁴ cells per well in 1% Geltrex‐coated 24‐well cell culture plates (SPL Life Sciences, 30024) in mTeSR1 supplemented with Y‐27632 dihydrochloride (10 µm) (Tocris Bioscience, 1254). The medium was replaced with mTeSR1 the following day. When the cells reached 40–60% confluence, they were washed with phosphate‐buffered saline (PBS) and treated with 8 µm CHIR (Sigma–Aldrich, SML1046) and 10 ng/ml Noggin (Peprotech, 120‐10C) in basic differentiation medium composed of advanced RPMI 1640 (Thermo Fisher Scientific, 12633‐020) and 100 × GlutaMAX (Thermo Fisher Scientific, 35050–061) for four days. On day 4, 10 ng mL^−1^ Activin A (R&D Systems, 338‐AC) was added for three days, followed by the addition of 10 ng mL^−1^ FGF9 (Peprotech, 100–23) and incubation for seven days. 3 µm CHIR was added from day 9 to 11. From day 14, only basic differentiation medium was given and replaced every three days. When renal vesicle‐like morphology was observed on day 10, CHIR was removed, and basic differentiation medium supplemented with 10 ng mL^−1^ FGF9 was used. For hypoxic conditions, cells were transferred into a hypoxic incubator at 37 °C (Eppendorf, New Brunswick Galaxy 48 R) with 1% or 5% O_2_–5% CO_2_–N_2_ balance continuously. To mimic hypoxia using CoCl_2_, cells were exposed to 100 µM CoCl_2_ (Sigma–Aldrich, C8661) from days 0 to 9 in an incubator at 37 °C with 5% CO_2_.

### Induction and Inhibition of Cyst Formation

To induce cysts in kidney organoids, the previously described basic differentiation medium was supplemented with 10 or 30 µm forskolin (LC Laboratories, F‐9929) every day from day 21. To inhibit cyst formation, 30 µm forskolin was administered on day 21 for 48 h, followed by treatment with either 1 or 10 µm niclosamide ethanolamine (Sigma–Aldrich, 5.33087) or 50 or 100 µm CFTRinh‐172 (MedChemExpress, HY‐16671) together with 30 µm forskolin for 24 h. Organoids were imaged using THUNDER Imaging Systems (Leica Microsystems), and their sizes were measured using ImageJ (NIH).

### Cisplatin‐Induced Nephrotoxicity Assay

Kidney organoids were exposed to 5 or 50 µm cisplatin (Sigma–Aldrich, P4394) on day 21. After 24 h, organoids were then fixed with 4% PFA and stained with anti‐KIM‐1, LTL, and CDH1 antibodies. The percentage area of KIM‐1 and LTL was measured using ImageJ (NIH).

### Immunofluorescence Microscopy

Cells were fixed with 4% paraformaldehyde (PFA) in PBS for 2 h at room temperature or overnight at 4 °C. Fixed cells were washed with PBS and blocked in blocking buffer [3 vol% fetal bovine serum (FBS), 1 wt.% bovine serum albumin (BSA), 0.5 vol% Triton X‐100 (Sigma–Aldrich, T8787), and 0.5 vol% Tween 20 (Sigma–Aldrich, P1379) in PBS] for 2 h. The antibodies used for immunostaining are listed in Table S3 (Supporting Information). Cells were incubated with primary antibodies diluted in the same buffer for 3 to 5 days at 4 °C, and then washed in PBST [0.1 vol% Triton X‐100 in PBS]. Secondary antibodies conjugated with Alexa Fluor‐488, 555, 647, or Streptavidin conjugated Cy5 in the same buffer were used for staining overnight at 4 °C. Cells were washed with PBST and then incubated for 2 h at room temperature in PBS with 2 µg mL^−1^ DAPI (Sigma–Aldrich, D9542). Cells were imaged using an LSM980 confocal microscope (Zeiss). Quantification of co‐localization, percentage area of the nephron segments, tubular length, and cyst volume was performed using ImageJ.

### Histology

Cystic organoids were fixed with 4% PFA in PBS for 2 h at room temperature. After fixation, the organoids were rinsed with PBS and embedded in paraffin using the STP120 tissue‐processor (Thermo Fisher Scientific). Paraffin blocks were sectioned into 5 µm slices using the Leica Microtome RM2255 (Leica Microsystems). The sections were deparaffinized, rehydrated, and stained with hematoxylin and eosin (H&E). Finally, the samples were mounted using Micromount (Leica Microsystems, 3801731) to acquire images.

### qRT‐PCR

mRNA expression was analyzed using qRT‐PCR. An AccuPrep Universal RNA Extraction Kit (Bioneer, K‐3140) was used to extract total RNA. For cDNA synthesis, an AccuPower RocketScript Cycle RT PreMix (Bioneer, K‐2202) and a T100 Thermal Cycler (Bio‐Rad) were used. qRT‐PCR was performed using SYBR Green Realtime PCR Master Mix (TOYOBO, QPK‐201) on a CFX Connect Real‐Time PCR Detection System (Bio‐Rad). All samples were run in two technical replicates and normalized with respect to GAPDH levels using the 2‐ΔΔCt method. The primer sequences are listed in Table S4 (Supporting Information).

### Bulk RNA‐Sequencing

Total RNA was isolated from kidney organoids on day 21 using TRIzol. Sequencing libraries were prepared using a TruSeq Stranded mRNA Library Prep Kit (Illumina). Sequencing was performed using a NovaSeq 6000 system (Illumina). Sequence reads were extracted in FASTQ format and trimmed from both ends based on a minimum read length of 36 bp. Reads were mapped to the human genome hg38 using HISAT2 v.2.1.0., and transcripts per kilobase million (TPM) were obtained using StringTie v.1.2.3b. Bulk RNA sequencing was commercially commissioned by Macrogen (Macrogen Inc.). Volcano plots and heat maps were generated using normalized gene expression represented by log_2_(TPM+1) and z‐scores, respectively. KEGG pathway and GO term enrichment analyses were performed using the DAVID database. Plots were generated using GraphPad Prism 9 and OriginPro.

### Single Cell RNA‐Sequencing

To obtain single cells from kidney organoids, detached organoids were harvested by gentle pipetting and then dissociated using Accutase (Sigma–Aldrich, A6964) for 20 min. Isolated cells were prepared according to manufacturer's protocol (10X Genomics). Single‐cell RNA sequencing was commercially commissioned by Ebiogen (Ebiogen Inc.). Briefly, sequencing libraries were constructed using 10X Chromium Next GEM Single Cell 3′ reagent kit v3.1 (10X Genomics). Raw sequencing data of FASTQ files were performed quality control and aligned to GRCh38 human reference genome using Cell Ranger (10X Genomics).

### Single Cell RNA‐Sequencing Data Analysis

The output files of Cell Ranger were analyzed with Seurat^[^ ^64^ ^]^ R package following the previous approach.^[^ ^22^ ^]^ Cells expressing less than 200 genes, more than 6000 genes, or more than 15% of mitochondrial genes were filtered out in each dataset. Obtained dataset of total 35281 cells from the normoxic (17163 cells) and hypoxic (18118 cells) conditions were normalized, and mitochondrial genes were regressed out using SCTransform.^[^ ^65^ ^]^ Identified 2000 highly variable features were dimensionally reduced by PCA and UMAP. Sub‐clustering of nephron cells (*MAFB* ^+^ or *EPCAM* ^+^) were performed using the same approach described above. Obtained 14 clusters in each subset data were annotated based on differential gene expression testing. For the comparison of gene expression, each cell type cluster was integrated using the canonical correlation analysis (CCA) method, and gene expression was visualized with dot plots. Reference‐based mapping was also implemented following the workflow by Satija lab. Cell types were predicted using the TransferData function in Seurat. The healthy human adult kidney datasets used as a reference in this study were generated by the Kidney Precision Medicine Project. The data were accessed on April 3, 2025, and are available at: https://www.kpmp.org.

### Scanning Electron Microscopy

Organoids were fixed using 2.5% glutaraldehyde in 0.1 m sodium cacodylate buffer for 1 h and washed for 2 min in PBS. Samples were further fixed with 1% osmium tetroxide (Sigma–Aldrich, 75633) for 1 h and washed with distilled water. Serial dehydration was performed in 25%, 50%, 75%, and 95% ethanol solutions and absolute ethanol for 3 min. After dehydration, hexamethyldisilazane (Sigma–Aldrich, 440191) was used. The samples were coated with platinum using sputter (Hitachi) and imaged using SU8220 (Hitachi). ImageJ was used to measure the lengths of primary cilia.

### Dextran Uptake Assay

Kidney organoids were incubated in basic differentiation medium with 100 µg mL^−1^ of 10000‐MW cascade blue‐labeled dextran (Thermo Fisher Scientific, D1976) for 24 h and then cultured in fresh basic differentiation medium for 24 h, following previous approaches.^[^ ^3a,e^ ^]^ Organoids were fixed with 4% PFA and stained with anti‐LTL antibody.

### Sodium Uptake Assay

Kidney organoids were incubated with 50 µm ouabain in sodium uptake assay buffer (90 mm NaCl, 60 mm N‐methyl‐d‐glucamine, 2 mm NaH_2_PO_4_, 5 mm KCl, 1 mM CaCl_2_, 1.2 mm MgSO_4_, 32 mm HEPES, and 10 mm glucose, pH 7.4). After 24 h, organoids were treated with 20 µm Sodium Green Tetraacetate (Thermo Fisher Scientific, S6901) for 3 h at room temperature. And then organoids were fixed with 4% PFA and stained with anti‐CDH1 antibody.

### Western Blot

Cells were lysed in 1 m Tris‐HCl, pH 6.8, 8 wt.% sodium dodecyl sulphate (SDS), 40 vol% glycerol containing beta‐mercaptoethanol, and bromophenol blue and separated on an SDS–polyacrylamide gel. Proteins in the gel were transferred onto a polyvinylidene fluoride membrane (Thermo Fisher Scientific, 88520) and then blocked with 3 wt.% BSA. The blots were incubated in PBS containing 0.1% Tween‐20 and 3% BSA with anti‐HIF1α antibody (1:1000, Novus Biologicals, AF1935) or anti‐beta actin antibody (1:1000, Abcam, ab8227) and then incubated with HRP‐conjugated anti‐rabbit (1:3000, Thermo Fisher Scientific, 65–6120) and anti‐goat (1:5000, Santa Cruz Biotechnology, sc‐2020) antibodies. Protein bands were visualized using a Pierce ECL Western Blotting Substrate (Thermo Fisher Scientific, 32106).

### Statistical Analysis and Reproducibility

All data represent mean ± standard error (S.E.). Statistical analysis was performed using Student's *t*‐test, one‐way, or two‐way analysis of variance (ANOVA) with Tukey's multiple comparison test or Šídák's multiple comparison test using GraphPad Prism 9; ^*^ *p* < 0.05; ^**^ *p* < 0.01; ^***^ *p* < 0.001; ^****^ *p* < 0.0001.

## Conflict of Interest

The authors declare no conflict of interest.

## Author Contributions

H.L. designed the study, conducted overall data acquisition, analysis, and visualization with T.‐E.P. and D.S.K., who supervised all work. D.K. contributed to analyzing the data. H.Y. contributed to the western blot experiments, and Y.J.K. supported protocol validation. J.H.K. contributed to scanning electron microscopy. H.L. and T.‐E.P. prepared the manuscript with input from all others.

## Supporting information



Supporting Information

## Data Availability

Single‐cell RNA sequencing data collected in this study have been deposited at the National Center for Biotechnology Information BioProjects Gene Expression Omnibus (GEO) under accession code GSE252639. This study uses referenced sources of code; no new code generated.
