# Supplementary material for: Developmental Hypoxia Enhances Kidney Organoid Complexity and Maturity
Source: Adv Sci (Weinh). 2025 Aug 21;12(40):e01661. doi: 10.1002/advs.202501661 (PMC12561403; doi:10.1002/advs.202501661)
Supplement: Supplementary file 1 — Supporting Information [file ADVS-12-e01661-s001.docx]

Supporting Information

Developmental hypoxia enhances kidney organoid complexity and maturity

Hyeonji Lim^1^, Dohui Kim^2^, Haejin Yoon^3^, Joo H. Kang^1^, Yong Jun Kim^4^, Dong Sung Kim^2,5,6,7,*^, Tae-Eun Park^1,*^

^1^ Department of Biomedical Engineering, College of Information‑Bio Convergence Engineering, Ulsan National Institute of Science and Technology (UNIST), Ulsan 44919, Republic of Korea.

^2^ Department of Mechanical Engineering, Pohang University of Science and Technology (POSTECH), Pohang 37673, Republic of Korea.

^3^ Department of Biological Sciences, College of Information‑Bio Convergence Engineering, Ulsan National Institute of Science and Technology (UNIST), Ulsan 44919, Republic of Korea.

^4^ Department of Pathology, College of Medicine, Kyung Hee University, Seoul 02447, Republic of Korea

^5^ Department of Chemical Engineering, Pohang University of Science and Technology (POSTECH), Pohang 37673, Republic of Korea.

^6^ School of Interdisciplinary Bioscience and Bioengineering, Pohang University of Science and Technology (POSTECH), Pohang 37673, Republic of Korea.

^7^ Institute for Convergence Research and Education in Advanced Technology, Yonsei University, Seoul 03722, Republic of Korea.

^*^ E-mail: smkds@postech.ac.kr (D.S.K) and tepark@unist.ac.kr (T.E.P)

**Table of Contents**

**Supplementary Figure3**

Figure S1. Differentiation of kidney organoids depending on oxygen conditions. 3

Figure S2. Maturation of kidney organoids differentiated under hypoxia. 4

Figure S3. Differentially expressed genes in kidney organoids under the hypoxia versus normoxia conditions.5

Figure S4. Tubular morphology of kidney organoids differentiated in the normoxic and hypoxic conditions. 7

Figure S5. Hypoxia-enhanced kidney organoids differentiated from WTC-11 cell lines exhibiting tubular improvement across cell lines. 8

Figure S6. The effect of hypoxia in extended culture on the development of highly structured tubules. 9

Figure S7. Identification of cell types in kidney organoids differentiated in the normoxic and hypoxic conditions at the single cell level. 10

Figure S8. Comparative analysis of cell types between kidney organoids and adult human kidney cell populations. 12

Figure S9. qRT-PCR analysis of AKT signaling regulatory genes under hypoxia.13

Figure S10. Immunostaining analysis of progenitor markers on day 9 in the hypoxia mimetic conditions. 14

Figure S11. Validation of cyst induced PKD models in the hypoxic and normoxic conditions.15

Figure S12. Cysplatin-induced injury in kidney organoids cultured under normoxia and hypoxia.16

**Supplementary Table17**

Table S1. Count of total cells in kidney organoids differentiated under normoxia and hypoxia. 17

Table S2. Count of sub-clustered cells in kidney organoids differentiated under normoxia and hypoxia. 18

Table S3. List of antibodies for immunostaining. 19

Table S4. List of primer sequences for qRT-PCR. 20


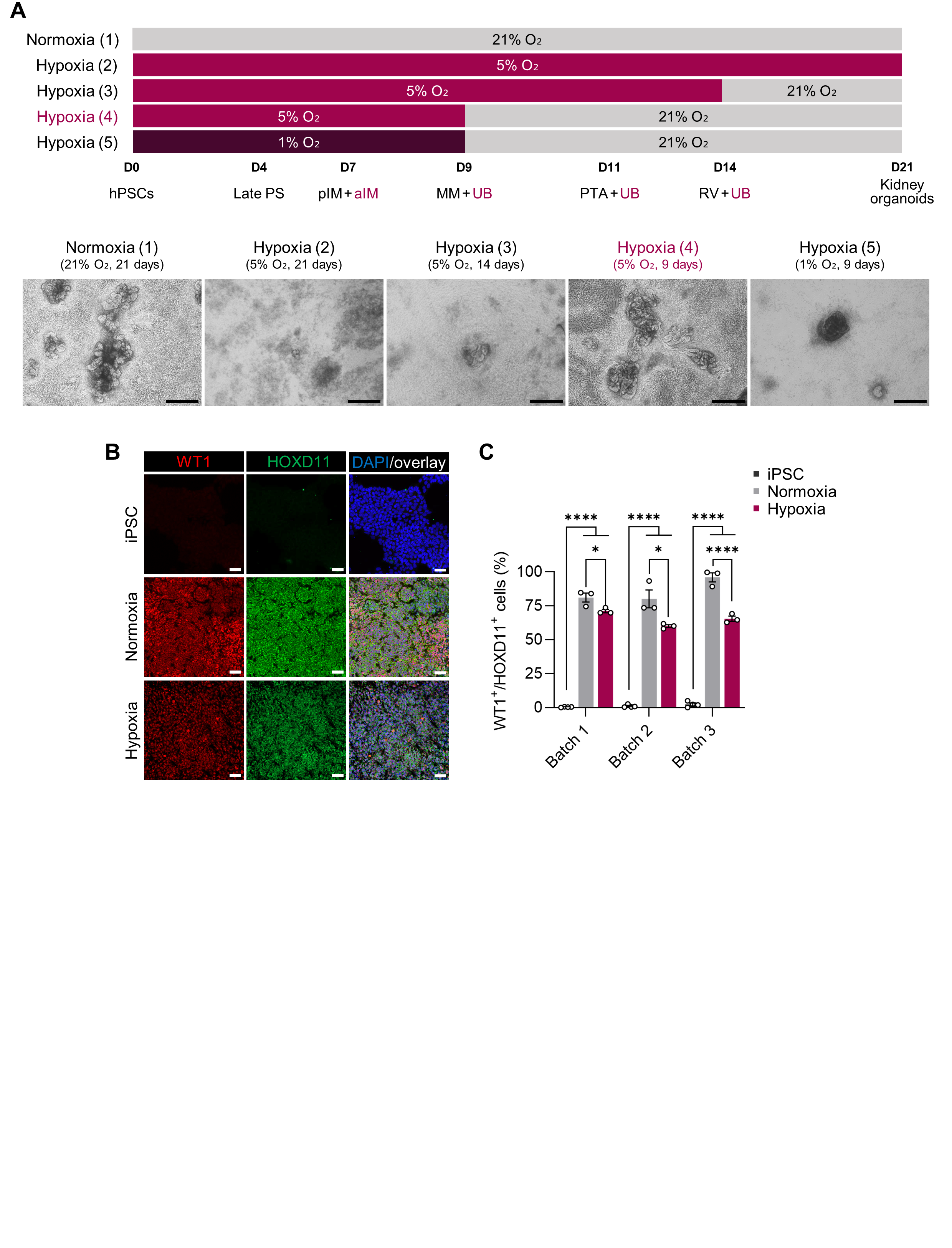


**Figure S1.** **Differentiation of kidney organoids depending on oxygen conditions.** (A) Timelines and corresponding bright field images of kidney organoids differentiated in each oxygen tension condition on day 21. Scale bars, 500 µm. hPSCs, human induced pluripotent stem cells; PS, primitive streak; pIM, posterior intermediate mesoderm; aIM, anterior intermediate mesoderm; MM, metanephric mesenchyme; UB, ureteric bud; PTA, pre-tubular aggregate; RV, renal vesicle. (B) Immunofluorescence micrographs of the iPSCs and cells on day 7 of differentiation labeled with pIM markers (WT1 and HOXD11). Scale bars, 50 µm. (C) The percentage of co‑expressed cells positive for WT1^+^/HOXD11^+^. *P* values were determined by one-way ANOVA followed by Tukey’s multiple comparison test (**P* < 0.05; *****P* < 0.0001).


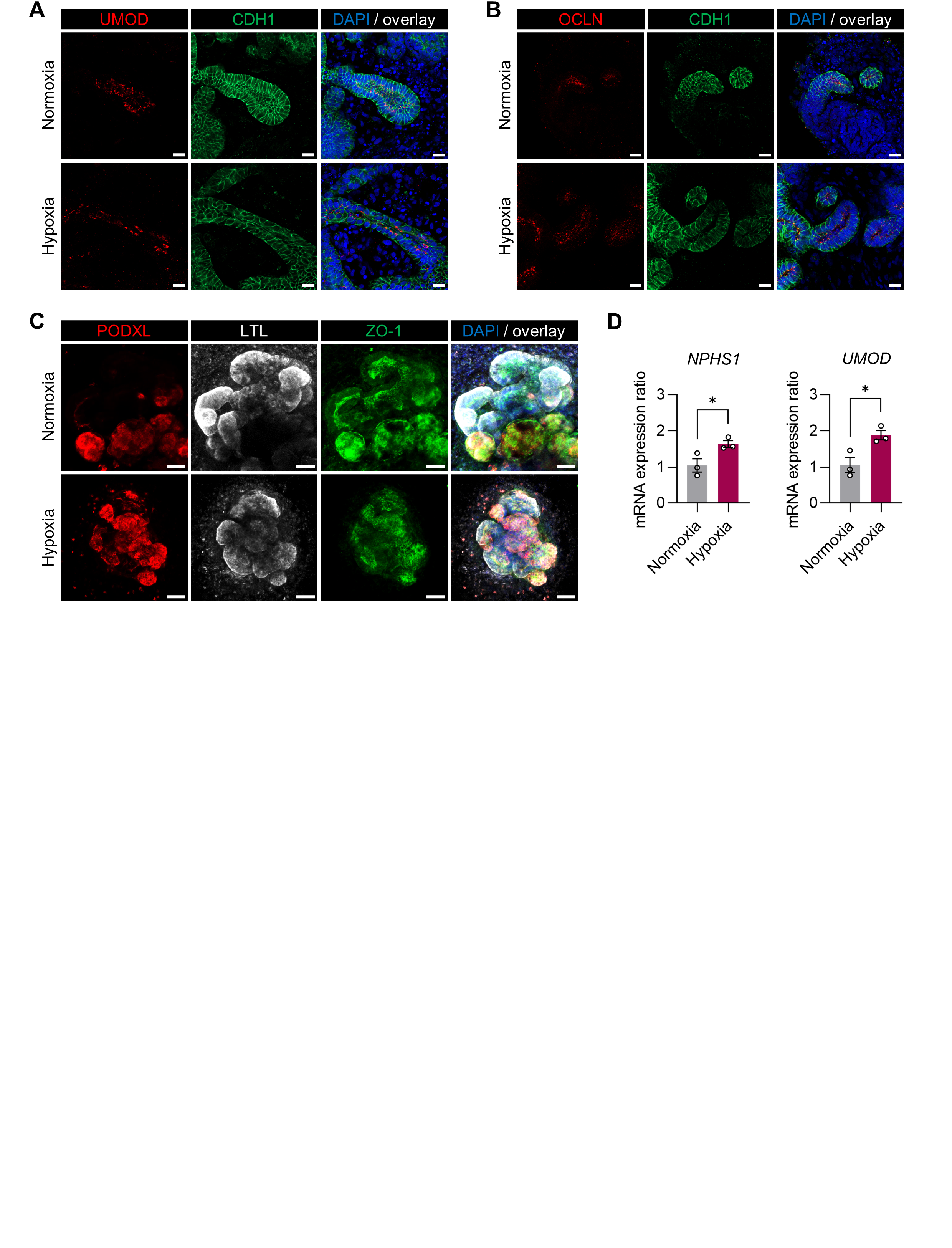


**Figure S2.** **Maturation of kidney organoids differentiated under hypoxia.** (A-C) Fluorescence microscopy images of kidney organoids, stained for a marker of loops of Henle (UMOD), loops of Henle/distal tubules/collecting ducts (CDH1), tight junction (OCLN), podocytes (PODXL), tight junction (ZO-1), and proximal tubules (LTL) on day 21-26 of differentiation under the normoxia and hypoxia conditions. Scale bars, 20 µm (A, B) and 100 µm (C). (D) The ratio of mRNA expression of genes encoding glomerular filtration barrier (*NPHS1*) and expressed in loops of Henle (*UMOD*). All data are plotted as mean ± S.E. and *N* = 3 for the independent experiments. Statistical test: Two-tailed unpaired t-test. (**P* < 0.05).

**
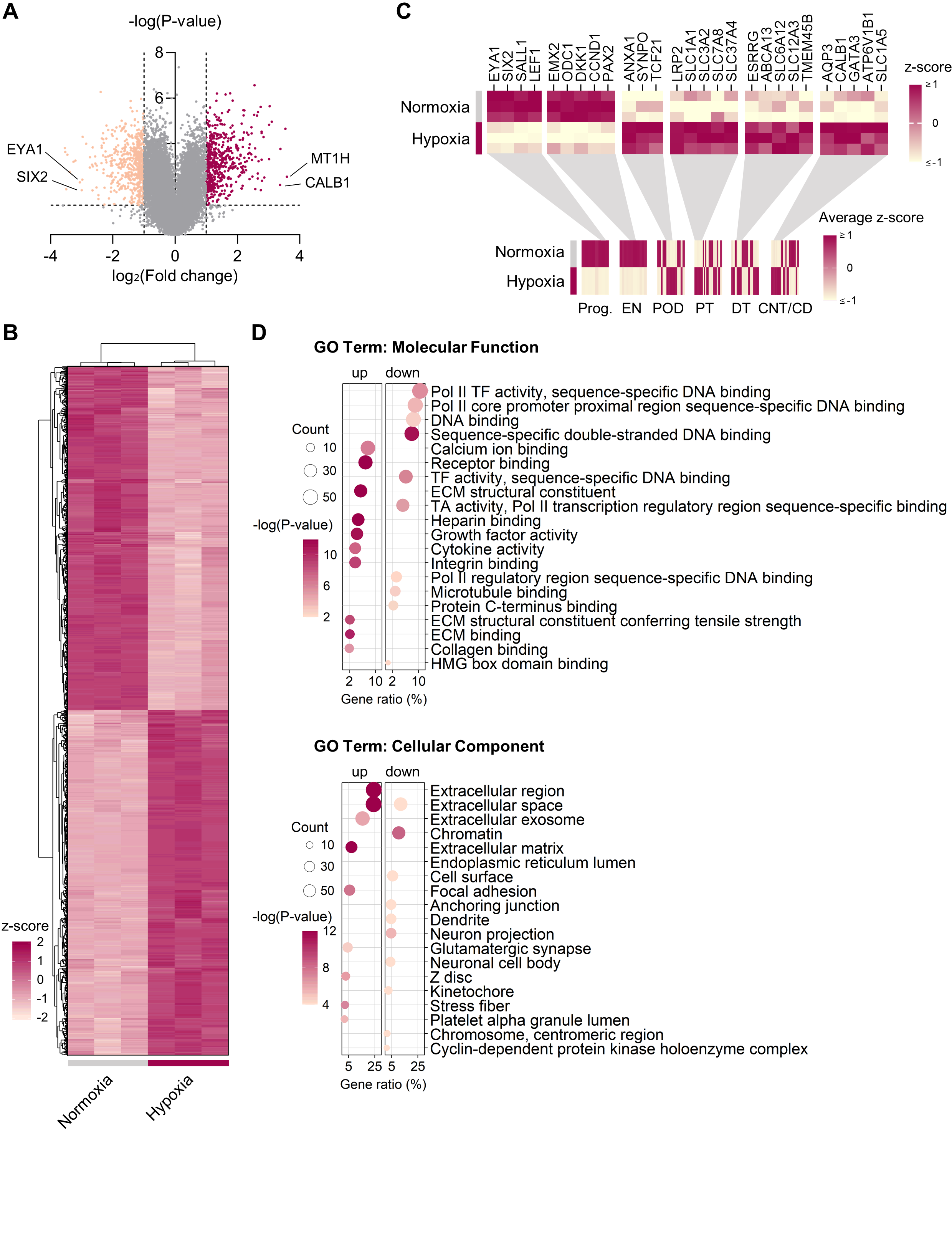
**

**Figure S3. Differentially expressed genes in kidney organoids under the hypoxia versus normoxia conditions.** (A) Volcano plot of the differentially expressed genes (DEGs) in kidney organoids on day 21. DEGs with |log_2_(fold change)| > 1 and *P* < 0.05 were marked in magenta or peach. (B) Heatmap of DEGs with |log_2_(fold change)| > 1 and *P* < 0.05 in the hypoxic and normoxic conditions. (C) Heatmap and hierarchical clustering of the selected DEGs expressed in each segment of kidney. Prog, progenitor; EN, early nephron; POD, podocyte; PT, proximal tubule; DT, distal tubule; CNT, connecting tubule; CD, collecting duct. (D) Molecular function and cellular component GO Terms of the kidney organoids under the hypoxia compared to normoxia. GO Terms on Y axis are ordered by gene ratio on X axis. The color gradient and size of the dots represent significance and the number of affected genes, respectively. Pol II, RNA polymerase II; TF, Transcription factor; TA, Transcriptional activator. All data are *N* = 3 for independent experiments.


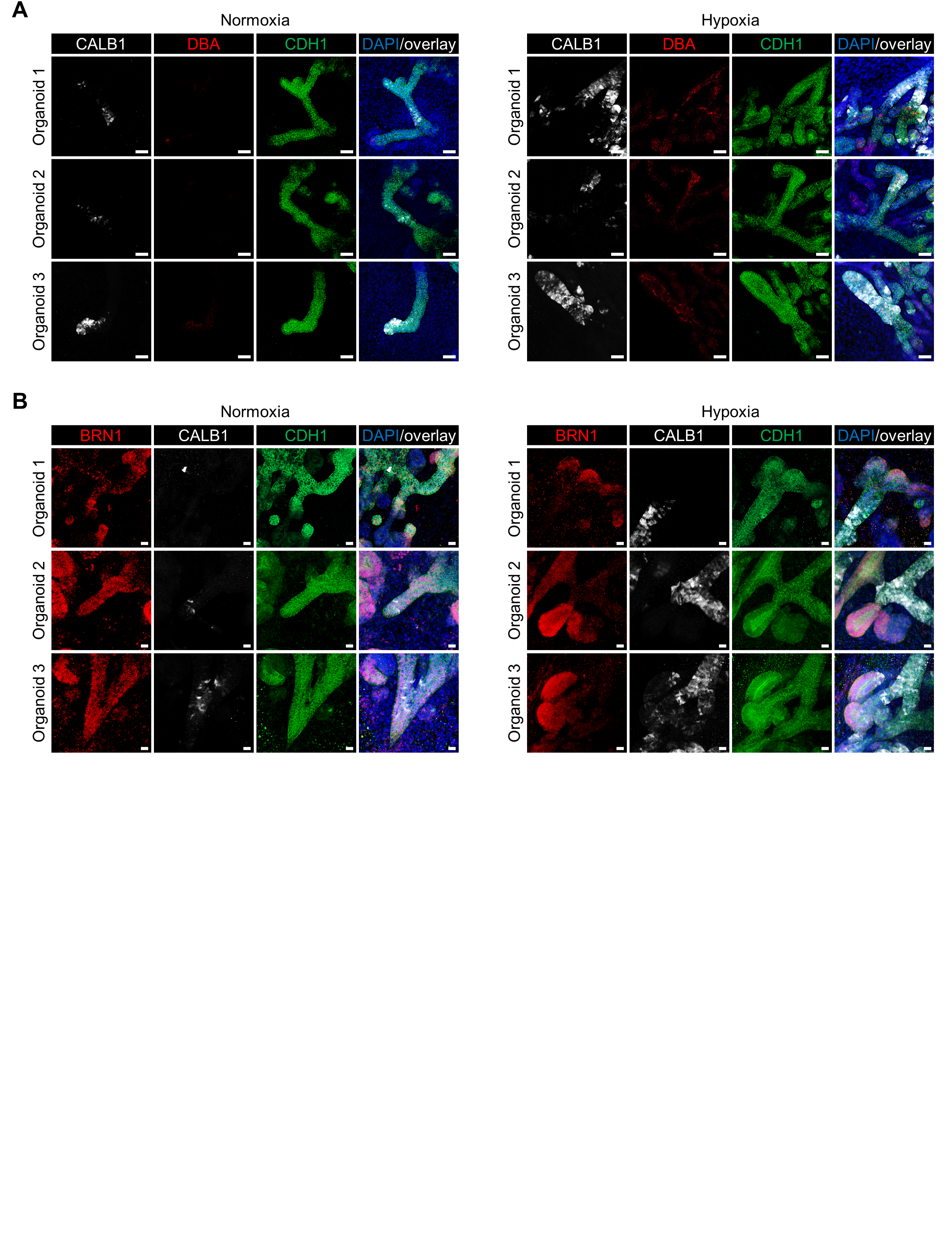


**Figure S4. Tubular morphology of kidney organoids differentiated in the normoxic and hypoxic conditions.** (A and B) Immunofluorescence micrographs of the kidney organoids on day 24, labeled with markers for distal tubules (BRN1 and CDH1) and collecting ducts (CDH1, CALB1, and DBA), differentiated under the normoxia and hypoxia conditions. Scale bars, 50 µm (A) and 20 µm (B).

**
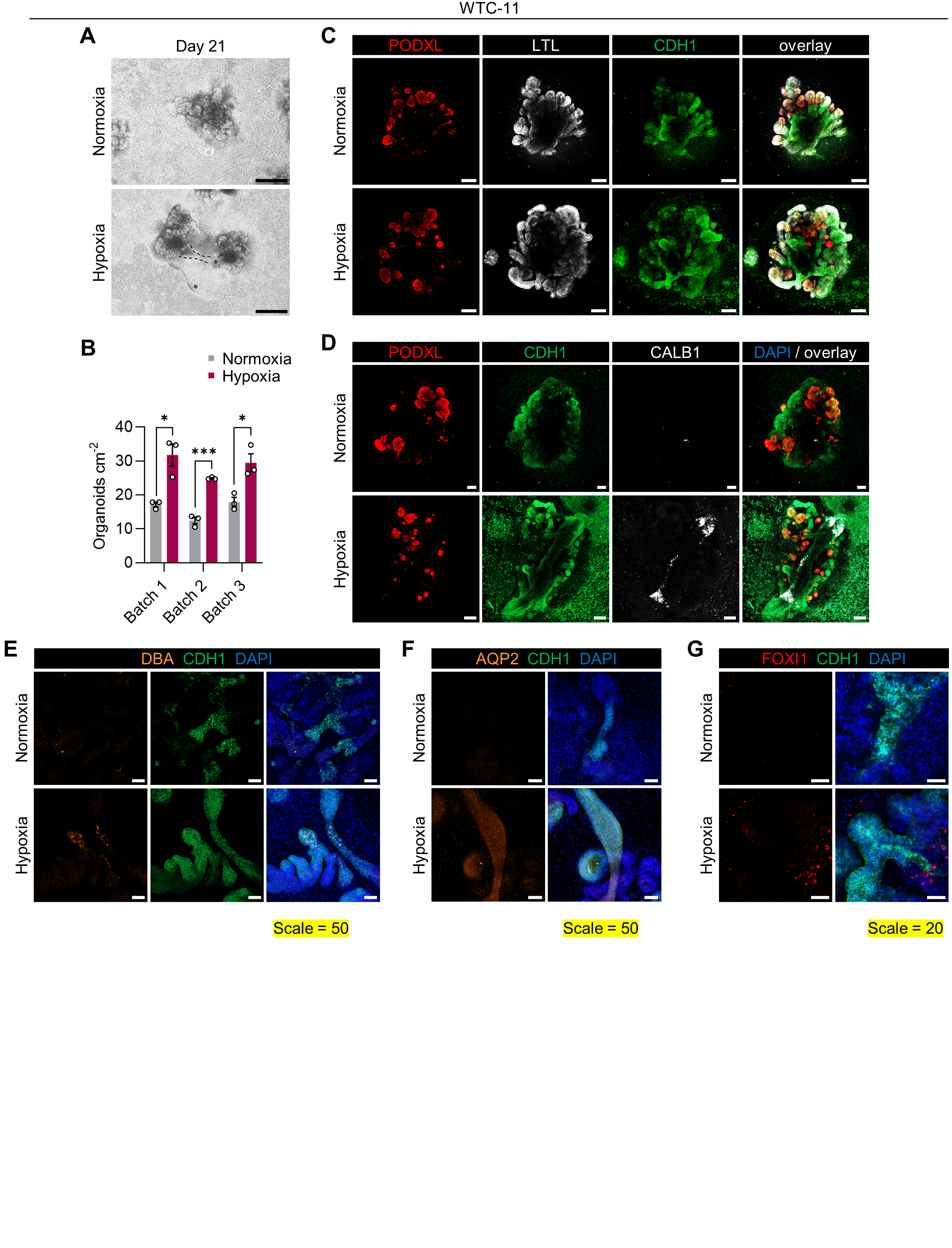
**

**Figure S5. Hypoxia-enhanced kidney organoids differentiated from WTC-11 cell lines exhibiting tubular improvement across cell lines.** (A) Bright-field images of kidney organoids on day 21 of differentiation in normoxia and hypoxia. Scale bars, 500 µm. (B) The number of kidney organoids > 200 µm per cm^2^ under normoxia and hypoxia conditions on day 21. All data are plotted as mean ± S.E. and *N* = 3 for the independent experiments. *P* values were determined by two-tailed unpaired t-test (**P* < 0.05; ****P* < 0.001). (C-G) Fluorescence microscopy images of WTC-11 (hiPSC line) derived-kidney organoids, stained for markers of podocyte (PODXL), proximal tubule (LTL), distal tubule/collecting duct (CDH1), and collecting duct (CALB1, DBA, AQP2, and FOXI1), on day 21-33 of differentiation under the normoxic and hypoxic conditions. Scale bars, 100 µm (C, D), 50 µm (E, F), and 20 µm (G).


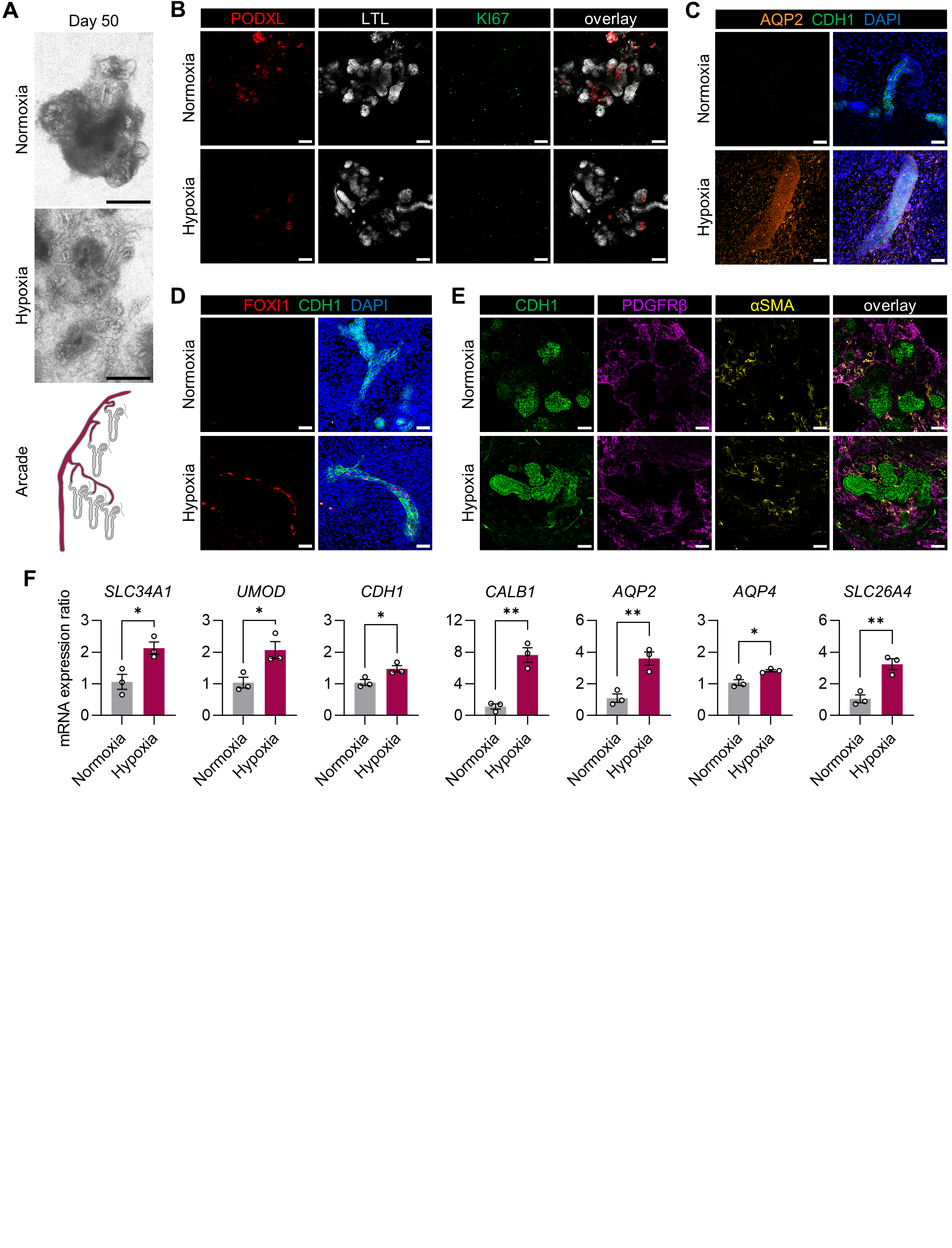


**Figure S6. The effect of hypoxia in extended culture on the development of highly structured tubules.** (A) Bright-field images of kidney organoids on day 50 of differentiation in the normoxic and hypoxic conditions. Scale bars, 500 µm. Highly branched tubules in hypoxia-enhanced kidney organoids resemble the renal arcade structure *in vivo*. (B-E) Immunofluorescence microscopy images of kidney organoids, stained for markers of podocyte (PODXL), proximal tubule (LTL), proliferation (KI67), loops of Henle/distal tubule/collecting duct (CDH1), collecting duct (AQP2 and FOXI1), mesenchyme (PDGFRβ), and myofibroblast (αSMA) on day 50 of differentiation in the normoxic and hypoxic conditions. Scale bars, 100 µm (B) and 50 µm (C-E). (F) The ratios of mRNA expression of genes encoding proximal tubule (*SLC34A1*), loop of Henle (*UMOD*), loops of Henle/distal tubule/collecting duct (*CDH1*), collecting duct (*CALB1*), principal cell (*AQP2* and *AQP4*) and intercalated cell (*SLC26A4*). All data are plotted as mean ±  S.E. and *N* = 3 for the independent experiments. *P* values were determined by two-tailed unpaired t-test (**P*< 0.05; ***P* < 0.01).

**
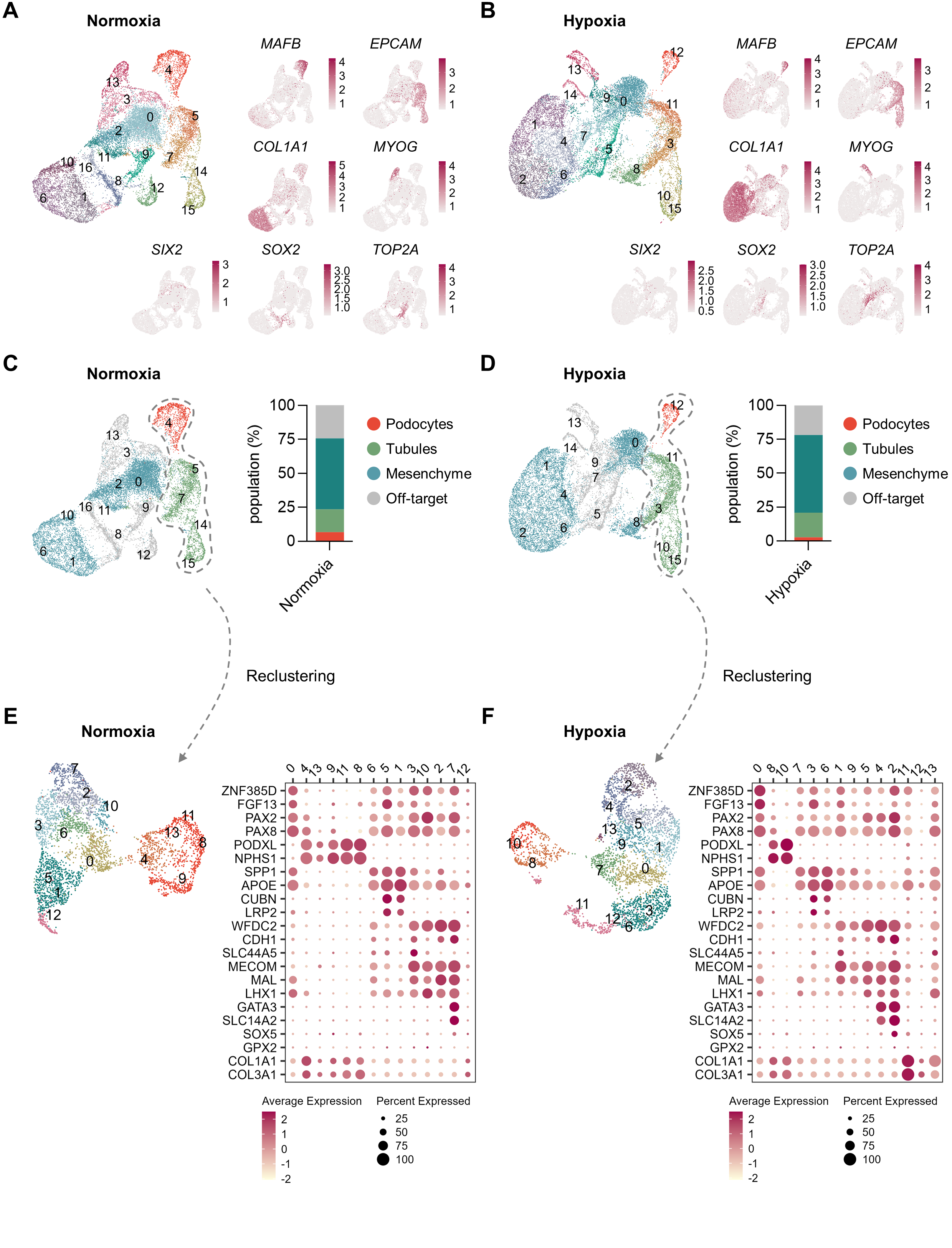
**

**Figure S7. Identification of cell types in kidney organoids differentiated in the normoxic and hypoxic conditions at the single cell level.** (A and B) Left: UMAP plots representing total cells from kidney organoids differentiated under normoxia (A) and hypoxia (B) on day 34. Right: UMAP plots showing the expression of cell type specific genes such as *MAFB* (podocytes), *EPCAM* (epithelial cells), *COL1A1* (mesenchymal cells), *MYOG* (muscle cells), *SOX2* (neural cells), and *TOP2A* (proliferating cells). (*N* = 1) (C and D) Left: UMAP plots showing identified clusters by the type of cells from kidney organoids cultured under normoxia (C) and hypoxia (D), each cell type represented in the same color. Right: Bar graphs displaying the proportion of each cell type. (E and F) Left: UMAP plots representing sub‑clustered nephron cells from kidney organoids differentiated under normoxia (E) and hypoxia (F). Right: Dot plots showing mRNA expression of key genes in each cluster.


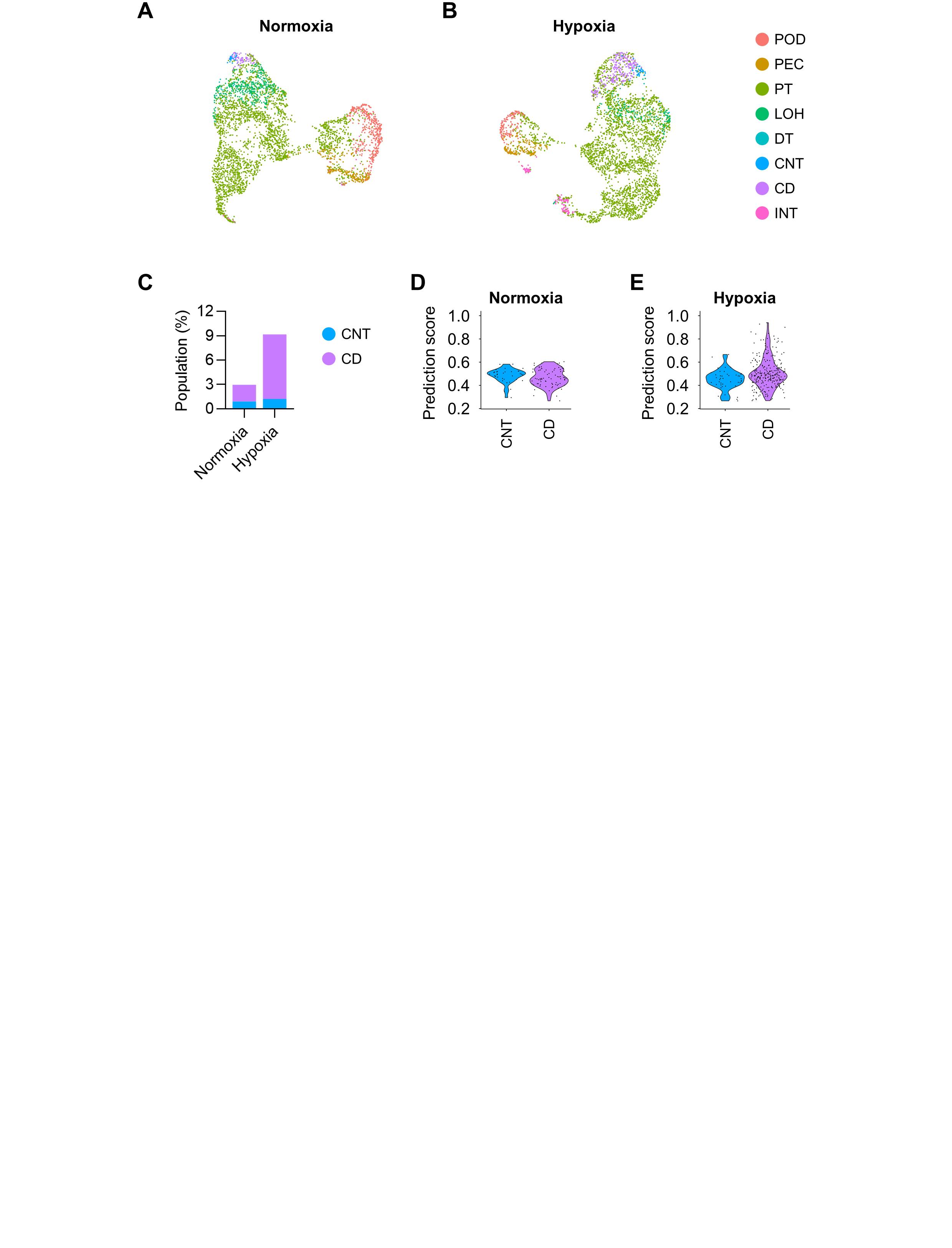


**Figure S8. Comparative analysis of cell types between kidney organoids and adult human kidney cell populations.** (A and B) UMAP plots of kidney organoid cells under normoxia (A) and hypoxia (B), mapped to reference datasets from human adult kidney. (C) Bar charts showing predicted cell populations in the CNT and CD clusters of kidney organoids under hypoxia compared to those under normoxia. (D and E) Violin plots representing mapping prediction scores for the CNT and CD clusters in kidney organoids under normoxia (D) and hypoxia (E). POD, podocyte; PEC, parietal epithelial cell; PT, epithelial cell of proximal tubule; LOH, kidney loop of Henle thin ascending limb epithelial cell; DT, kidney distal convoluted tubule epithelial cell; CNT, kidney connecting tubule epithelial cell; CD, kidney collecting duct principal cell; INT, kidney interstitial cell.

**
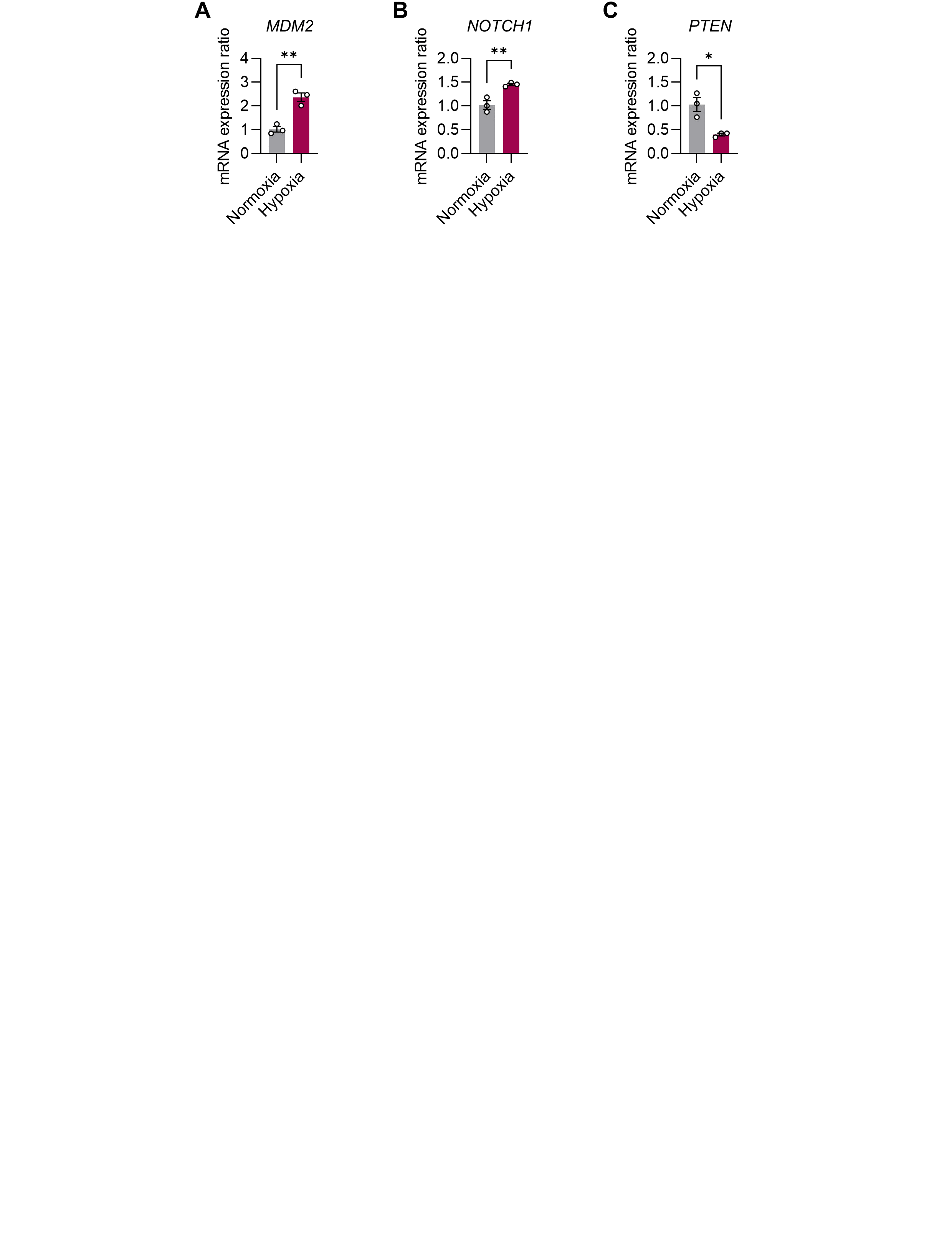
**

**Figure S9. qRT-PCR analysis of AKT signaling regulatory genes under hypoxia.** The mRNA expression ratios of the AKT regulatory genes, including *MDM2* (A), *NOTCH1* (B), and *PTEN* (C), on day 9. All data are plotted as mean ±  S.E. and *N* = 3 for the independent experiments. *P* values were determined by two-tailed unpaired t-test (**P*< 0.05; ***P* < 0.01).


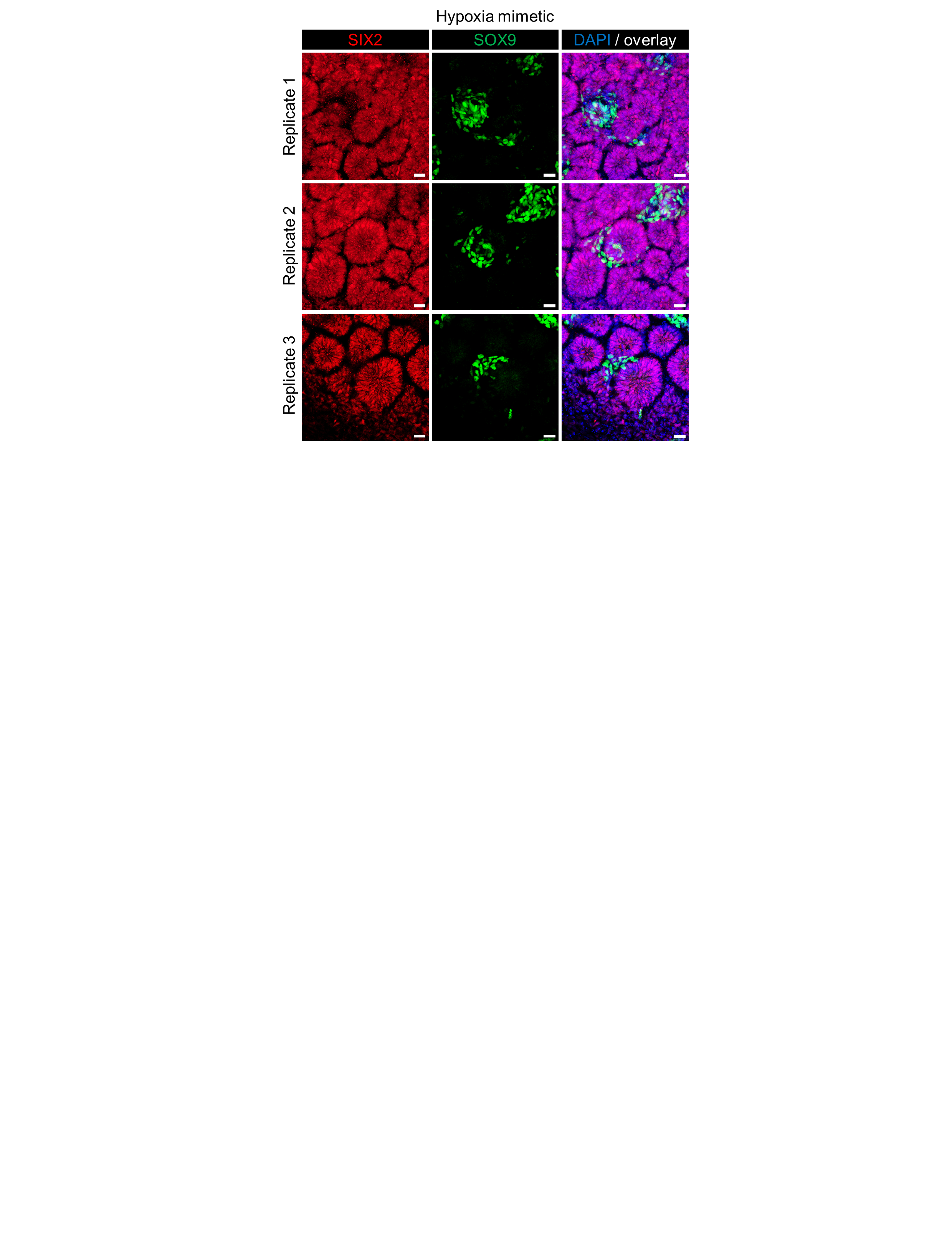


**Figure S10.** **Immunostaining analysis of progenitor markers on day 9 in the hypoxia mimetic conditions.** Immunofluorescence images of CoCl_2_‑treated cells showing expression of SIX2 and SOX9 on day 9. Scale bars, 20 μm.


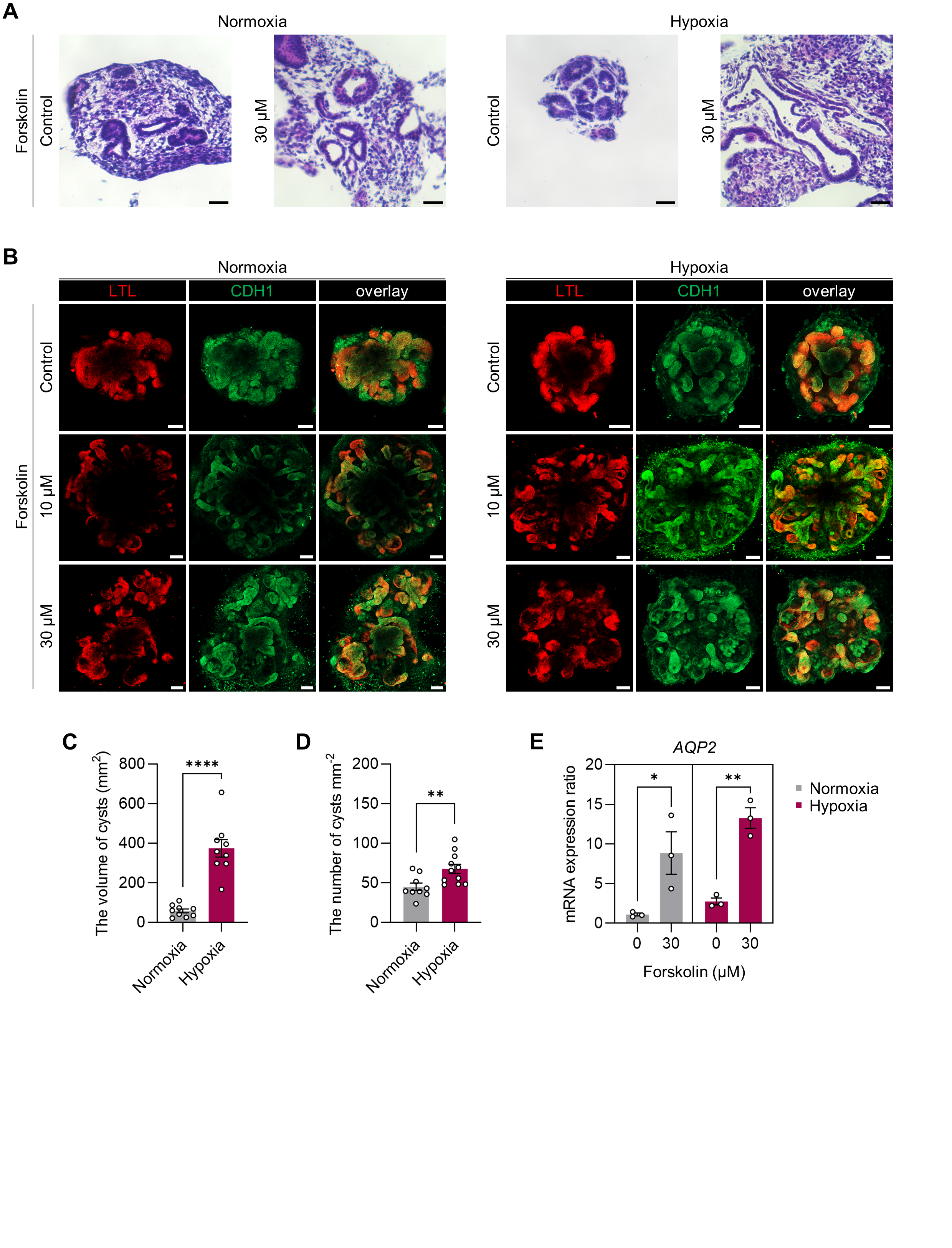


**Figure S11. Validation of cyst induced PKD models in the hypoxic and normoxic conditions.** (A) H&E images of control and cyst induced PKD organoids under normoxia and hypoxia. Scale bars, 50 µm. (B) Immunofluorescence microscopy images of cyst induced PKD organoids, labelled with markers of proximal tubule (LTL), and loop of Henle/distal tubule/collecting duct (CDH1), in the normoxic and hypoxic conditions. Scale bars, 100 µm. (C) The volume of individual cysts generated in 30 µM forskolin-induced PKD organoids under hypoxia compared to those under normoxia. (D) The number of counted cysts per mm^2^ in the normoxic and hypoxic kidney organoids treated with 30 μM forskolin. (E) The ratios of *AQP2* expression in kidney organoids cultured in the hypoxic versus normoxic conditions following treatment with 30 μM forskolin, compared to non‑treated organoids under normoxia. All data are plotted as mean ± S.E. and *N* = 3 for the independent experiments. *P* values were determined by (C, and D) two-tailed unpaired t-test and (E) two-way ANOVA followed by Tukey’s multiple comparison test (**P*< 0.05; ***P* < 0.01; *****P* < 0.0001).


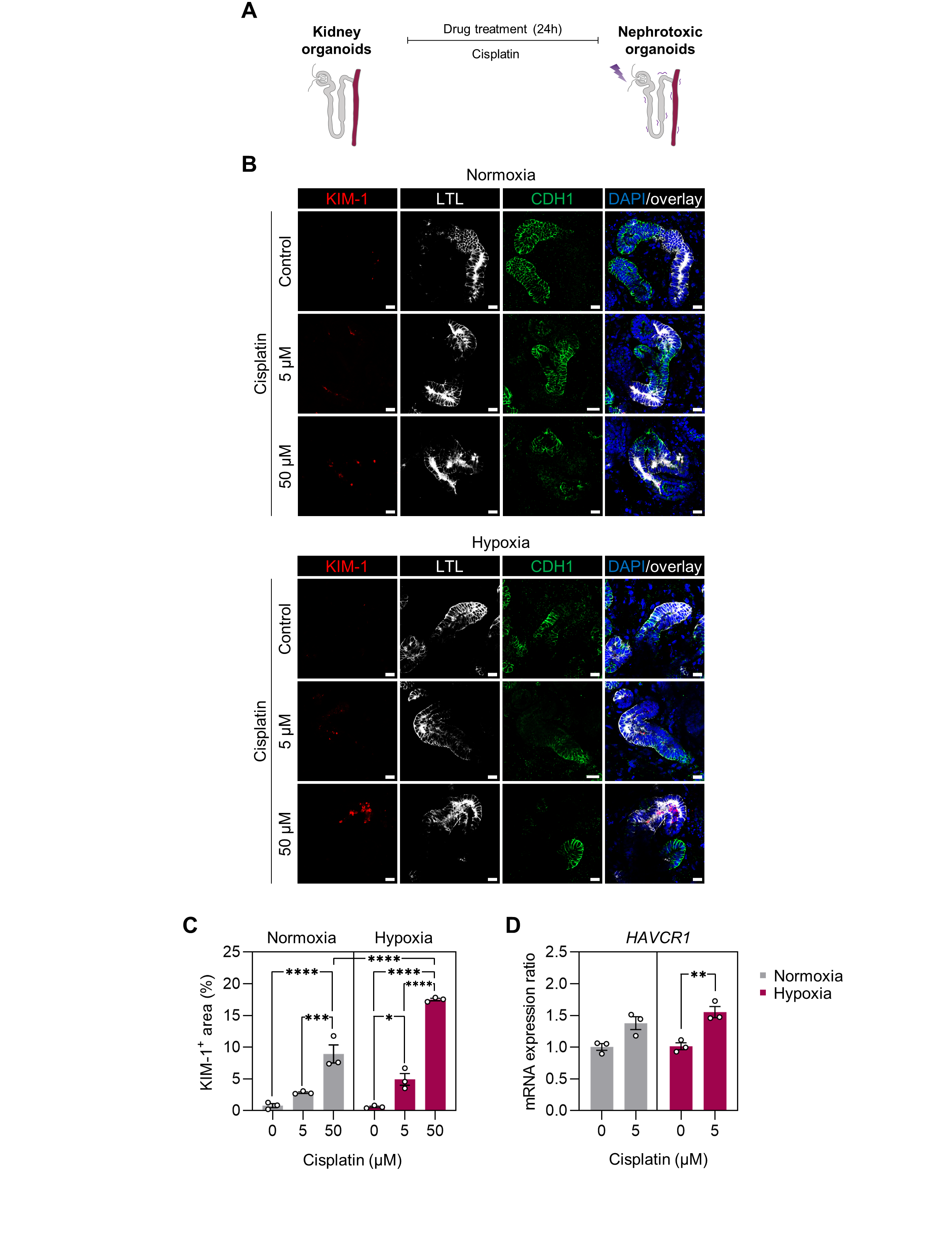


**Figure S12.** **Cisplatin-induced injury in kidney organoids cultured under normoxia and hypoxia.** (A) Schematic of the timeline for cisplatin exposure. (B) Immunofluorescence analysis of injured kidney organoids, exhibiting markers for kidney injury molecule-1 (KIM-1), proximal tubule (LTL), and loop of Henle/distal tubule/collecting duct (CDH1) under normoxia and hypoxia. Scale bars, 20 µm. (C) The percentage of KIM-1^+^ relative to LTL^+^ area of kidney organoids. (D) The ratios of *HAVCR1* expression of kidney organoids exposed to cisplatin compared to that non-treated control. All data are plotted as mean ± S.E. and *N* = 3 for the independent experiments. *P* values were determined by two-way ANOVA followed by (C) Tukey’s multiple comparison test and (D) Šídák's multiple comparison test (**P* < 0.05; ***P* < 0.01; ****P* < 0.001; *****P* < 0.0001).

**Table S1. Count of total cells in kidney organoids differentiated under normoxia and hypoxia.**

| **Normoxia** | | | **Hypoxia** | | |
| --- | --- | --- | --- | --- | --- |
| **Cluster** | **Cell type** | **Count** | **Cluster** | **Cell type** | **Count** |
| 0 | Mesenchyme | 3,924 | 0 | Mesenchyme | 3,132 |
| 1 | Mesenchyme | 1,510 | 1 | Mesenchyme | 2,018 |
| 2 | Mesenchyme | 1,273 | 2 | Mesenchyme | 1,856 |
| 3 | NPC | 1,191 | 3 | Tubule | 1,374 |
| 4 | Podocyte | 1,139 | 4 | Mesenchyme | 1,317 |
| 5 | Tubule | 1054 | 5 | Neuron | 1,164 |
| 6 | Mesenchyme | 971 | 6 | Mesenchyme | 1,071 |
| 7 | Tubule | 866 | 7 | Proliferating cell | 1,046 |
| 8 | Neuron | 770 | 8 | Mesenchyme | 959 |
| 9 | Proliferating cell | 731 | 9 | NPC | 917 |
| 10 | Mesenchyme | 664 | 10 | Tubule | 893 |
| 11 | Mesenchyme | 632 | 11 | Tubule | 879 |
| 12 | Neuron | 610 | 12 | Podocyte | 494 |
| 13 | Muscle | 602 | 13 | Muscle | 481 |
| 14 | Tubule | 582 | 14 | Neuron | 352 |
| 15 | Tubule | 396 | 15 | Tubule | 165 |
| 16 | Neuron | 248 | - | - | - |

**Table S2. Count of sub-clustered cells in kidney organoids differentiated under normoxia and hypoxia.**

| **Normoxia** | | | **Hypoxia** | | |
| --- | --- | --- | --- | --- | --- |
| **Cluster** | **Cell type** | **Count** | **Cluster** | **Cell type** | **Count** |
| 0 | EN | 472 | 0 | ET | 430 |
| 1 | PT | 423 | 1 | EDT | 392 |
| 2 | DT | 398 | 2 | CD | 351 |
| 3 | LOH | 382 | 3 | PT | 331 |
| 4 | EPOD | 346 | 4 | CNT | 319 |
| 5 | PT | 332 | 5 | DT | 315 |
| 6 | ET | 301 | 6 | PT | 313 |
| 7 | CNT | 283 | 7 | EN | 295 |
| 8 | POD | 282 | 8 | EPOD | 289 |
| 9 | POD | 262 | 9 | LOH | 265 |
| 10 | EDT | 174 | 10 | POD | 192 |
| 11 | POD | 160 | 11 | MS | 169 |
| 12 | MS | 126 | 12 | MS | 127 |
| 13 | POD | 96 | 13 | T/M | 17 |

**Table** **S3. List of antibodies for** **immunostaining.**

| **Antibody** | **Source** | **Catalog #** | **Concentration** |
| --- | --- | --- | --- |
| αSMA | Thermo Fisher Scientific | 14-9760-82 | 1:100 |
| AQP2 | Novus Biologicals | NB110-74682 | 1:100 |
| BRN1 | Novus Biologicals | NBP1-49872 | 1:100 |
| CALB1 | Sigma-Aldrich | C9848 | 1:100 |
| CDH1 | Proteintech | 20874-1-AP | 1:100 |
| CDH1 | abcam | ab11512 | 1:300 |
| DBA | Vector Laboratories | RL-1032-2 | 1:100 |
| FOXI1 | Novus Biologicals | NB300-926 | 1:300 |
| HIF1α | Novus Biologicals | AF1935 | 1:50 |
| HOXD11 | abcam | Ab55255 | 1:100 |
| KI67 | Santa Cruz Biotechnology | sc-23900 | 1:100 |
| KIM-1 | R&D Systems | AF1750 | 1:500 |
| LHX1 | Developmental Studies Hybridoma Bank | 4F2-c | 1:500 |
| LTL | Vector Laboratories | B-1325 | 1:200 |
| OCLN | Thermo Fisher Scientific | 33-1500 | 1:100 |
| PAX2 | Novus Biologicals | AF3364 | 1:200 |
| PAX8 | Proteintech | 10336-1-AP | 1:500 |
| PDGFRβ | R&D Systems | AF385 | 1:100 |
| PECAM1 | abcam | ab9498 | 1:100 |
| PODXL | R&D Systems | AF1658 | 1:200 |
| SIX2 | Proteintech | 11562-1-AP | 1:500 |
| SOX9 | R&D Systems | AF3075 | 1:100 |
| TBX6 | R&D Systems | AF4744 | 1:100 |
| UMOD | abcam | ab167678 | 1:100 |
| WT1 | Novus Biologicals | NBP2-67587 | 1:200 |
| ZO-1 | Thermo Fisher Scientific | 40-2200 | 1:100 |
| Donkey Anti-Goat IgG H&L (Alexa Fluor® 555) | abcam | ab150134 | 1:200 |
| Donkey Anti-Goat IgG H&L (Alexa Fluor® 647) | abcam | ab150135 | 1:200 |
| Donkey Anti-Mouse IgG H&L (Alexa Fluor® 488) | abcam | ab150109 | 1:200 |
| Donkey Anti-Rabbit IgG H&L (Alexa Fluor® 488) | abcam | ab150061 | 1:200 |
| Donkey Anti-Rabbit IgG H&L (Alexa Fluor® 647) | abcam | ab150063 | 1:200 |
| Streptavidin, Cy5 | Vector Laboratories | SA-1500-1 | 1:200 |

**Table S4. List of primer sequences for qRT-PCR.**

| **Gene** | **Forward** | **Reverse** |
| --- | --- | --- |
| *ABCB1* | GGGAGCTTAACACCCGACTTA | GCCAAAATCACAAGGGTTAGCTT |
| *ANO1* | CTGATGCCGAGTGCAAGTATG | AGGGCCTCTTGTGATGGTACA |
| *AQP1* | TAACCCTGCTCGGTCCTTTG | AGTCGTAGATGAGTACAGCCAG |
| *AQP2* | CTCCCTCCTCTACAACTACGTG | CTCCTCCCAATCGGTGTCC |
| *AQP4* | CATGGAAATCTTACCGCTGGT | TCAGTCCGTTTGGAATCACAG |
| *TBXT* | CTGGGTACTCCCAATGGGG | GGTTGGAGAATTGTTCCGATGA |
| *CALB1* | TCCAGGGAATCAAAATGTGTGG | GCACAGATCCTTCAGTAAAGCA |
| *CFTR* | TGCCCTTCGGCGATGTTTTT | GTTATCCGGGTCATAGGAAGCTA |
| *CDH1* | ATTTTTCCCTCGACACCCGAT | TCCCAGGCGTAGACCAAGA |
| *FGF10* | CAGTAGAAATCGGAGTTGTTGCC | TGAGCCATAGAGTTTCCCCTTC |
| *GAPDH* | TGTGGGCATCAATGGATTTGG | ACACCATGTATTCCGGGTCAAT |
| *GDNF* | GGCAGTGCTTCCTAGAAGAGA | AAGACACAACCCCGGTTTTTG |
| *HAVCR1* | TGGCAGATTCTGTAGCTGGTT | AGAGAACATGAGCCTCTATTCCA |
| *MDM2* | GAATCATCGGACTCAGGTACATC | TCTGTCTCACTAATTGCTCTCCT |
| *NOTCH1* | TCAGCGGGATCCACTGTGAG | ACACAGGCAGGTGAACGACTTG |
| *NPSH1* | GGCTCCCAGCAGAAACTCTT | CACAGACCAGCAACTGCCTA |
| *PECAM1* | AACAGTGTTGACATGAAGAGCC | TGTAAAACAGCACGTCATCCTT |
| *PTEN* | TTTGAAGACCATAACCCACCAC | ATTACACCAGTTCGTCCCTTTC |
| *SIX2* | GGCCAAGGAAAGGGAGAACA | GAGCTGCCTAACACCGACTT |
| *SLC26A4* | TGGTGGGATCTGTTGTTCTGA | GGATCTGCCAAGTACCTCACT |
| *SLC34A1* | TCACGAAGCTCATCATCCAG | TTCCTCAGGGACTCATCACC |
| *SLC4A1* | GGTGATGGACGAAAAGAACCA | AAGACTCTACGCAGCTCTAGG |
| *UMOD* | ATGTGGGGCCAATGACATGAA | CAGTCCCGGTTGTCTCTGT |
| *WNT4* | AGGAGGAGACGTGCGAGAAA | CGAGTCCATGACTTCCAGGT |
| *WNT9B* | TGTGCGGTGACAACCTCAAG | ACAGGAGCCTGATACGCCAT |
| *WNT11* | ATGTGCGGACAACCTCAGCTAC | GATGGAGCAGGAGCCAGACA |
